# Metabolite‐Based Network Pharmacology, Molecular Docking, and Dynamics Simulations to Preliminarily Verify Treating Diabetic Encephalopathy Effect of Kuwanon G

**DOI:** 10.1002/fsn3.70392

**Published:** 2025-06-07

**Authors:** Yuqian Zhang, Siying Zhang, Haiying Niu, Weiwei Xie, Yuxin Tan, Deqiang Li, Yiran Jin

**Affiliations:** ^1^ The Second Hospital of Hebei Medical University Shijiazhuang Hebei P. R. China; ^2^ The First Hospital of Hebei Medical University Shijiazhuang Hebei P. R. China; ^3^ Hebei Province Center for Disease Prevention and Control Shijiazhuang Hebei P. R. China

**Keywords:** Kuwanon G, metabolism, molecular docking, molecular dynamics simulation, neuroprotection

## Abstract

Kuwanon G (KWG), a bioactive flavonoid from mulberry, exhibits potential neuroprotective effects against diabetic encephalopathy (DE), yet its metabolic fate and therapeutic mechanisms remain unclear. This study integrated ultra‐high‐performance liquid chromatography–quadrupole time‐of‐flight mass spectrometry (UHPLC‐Q‐TOF‐MS), network pharmacology, molecular docking, and dynamics simulations to characterize KWG's metabolic profile and evaluate its anti‐DE activity. In vivo and in vitro analyses identified 56 metabolites in rats, predominantly formed via oxidation, dehydrogenation, methylation, and glucuronidation. Nine metabolites with high intestinal absorption and pharmacophore compatibility were selected using Swiss ADME. Network pharmacology revealed core targets (AKT1, TNF, SRC, EGFR, ESR1) linked to DE, while molecular docking demonstrated strong binding affinities (−4.87 to −43.41 kcal/mol) between active metabolites (N1, N4, N6, N8) and these targets. Dynamics simulations confirmed stable interactions, highlighting metabolites' roles in modulating PI3K‐Akt signaling and neurodegeneration pathways. Notably, KWG itself exhibited negligible binding, suggesting its metabolites are the primary bioactive forms. These findings underscore the importance of gut microbiota‐mediated biotransformation in enhancing KWG's bioavailability and neuroprotective efficacy. This work provides critical insights into the metabolic activation of natural products and advances their application in functional foods or therapeutics for diabetes‐related complications.

## Graphical abstract

This study investigates the neuroprotective effects of Kuwanon G (KWG) against diabetic encephalopathy (DE) by characterizing its metabolic profile and therapeutic mechanisms. Using UHPLC‐Q‐TOF‐MS, network pharmacology, molecular docking, and dynamics simulations, 56 metabolites were identified, with nine showing high intestinal absorption and pharmacophore compatibility. Key findings suggest that KWG's metabolites, particularly N1, N4, N6, and N8, modulate PI3K‐Akt signaling and neurodegeneration pathways, indicating their potential as therapeutic agents for DE.

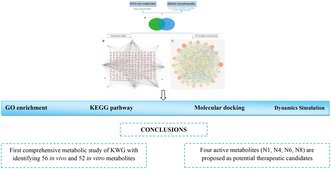

## 1 Introduction

**FIGURE 1:**
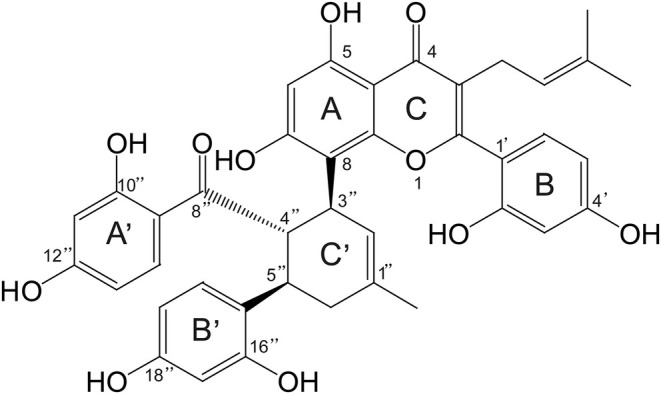
Chemical structure of KWG.

Mulberry (*Morus* spp.), a traditional agricultural crop cultivated for millennia in East Asia, has been widely used in sericulture and herbal medicine due to its diverse pharmacological properties (Li et al. 2020; He et al. 2013). Beyond its role as silkworm feed, mulberry leaves are recognized for their functional nutraceutical potential, offering antioxidant, anti‐inflammatory, and neuroprotective benefits (Park et al. 2021; Srivastava et al. 2009; Hunyadi et al. 2013; Hu et al. 2020). Kuwanon G (KWG, Figure 1), belonging to mulberry Diels–Alder‐type adducts (MDAAs) (Mei et al. 2012; Li et al. 2020; Park et al. 2021), is a bioactive constituent present in mulberry (Yuan and Zhao 2017; Chen, Zhang, et al. 2016; Zhou et al. 2017; Luo et al. 2022). Studies have shown that KWG has significant antioxidant stress (Guo et al. 2016), neuroprotective (Gan et al. 2021) and anti‐inflammatory effects (Paudel et al. 2018; Kuk et al. 2017).

Diabetic encephalopathy (DE) a severe complication of diabetes mellitus, is characterized by cognitive decline, memory impairment, and an increased risk of neurodegenerative disorders such as Alzheimer's disease (Markowicz‐Piasecka et al. 2017). The accumulation of advanced glycation end products (AGEs) is a critical factor in DE pathogenesis, yet effective therapeutic interventions remain limited (Gireesh et al. 2009). Current management strategies primarily focus on glycemic control, highlighting the urgent need for novel treatments (Saliu et al. 2016). Natural products, with their low toxicity and high safety profiles, have emerged as potential therapeutic agents for DE. KWG, in particular, has demonstrated neuroprotective effects by modulating the PI3K/Akt/GSK3αβ signaling pathway, which mitigates AGE‐induced neuronal damage (Zhu et al. 2018).

Despite growing evidence supporting the neuroprotective effects of KWG, significant gaps remain in elucidating its mechanism of action and therapeutic potential. Prior studies have predominantly concentrated on the pharmacological effects of the parent compound, while the metabolic fate of KWG and the bioactivity of its metabolites remain largely unexplored. This gap is particularly noteworthy, as natural products frequently exert their therapeutic effects via metabolites rather than their native forms—a phenomenon well established for flavonoids such as quercetin and resveratrol (Yu et al. 2012; Chen, Wen, et al. 2016; Zhang et al. 2020). To date, no systematic investigation has addressed whether KWG's neuroprotection is mediated by its biotransformation products. Additionally, existing studies on KWG have predominantly focused on single pathways, such as the PI3K/Akt signaling pathway (Gan et al. 2021), while overlooking the multifactorial nature of DE, which encompasses oxidative stress, neuroinflammation, and synaptic dysfunction. Consequently, this study seeks to address these limitations by comprehensively characterizing the metabolic profile of KWG, identifying bioactive metabolites, and elucidating their multi‐target interactions within DE‐related pathways. By integrating metabolic profiling, network pharmacology, and computational simulation, the objective is to redefine KWG not merely as a single‐molecule drug but as a prodrug capable of microbial metabolic activation, thereby offering novel insights into its application for treating diabetic neurodegenerative complications.

**FIGURE 2:**
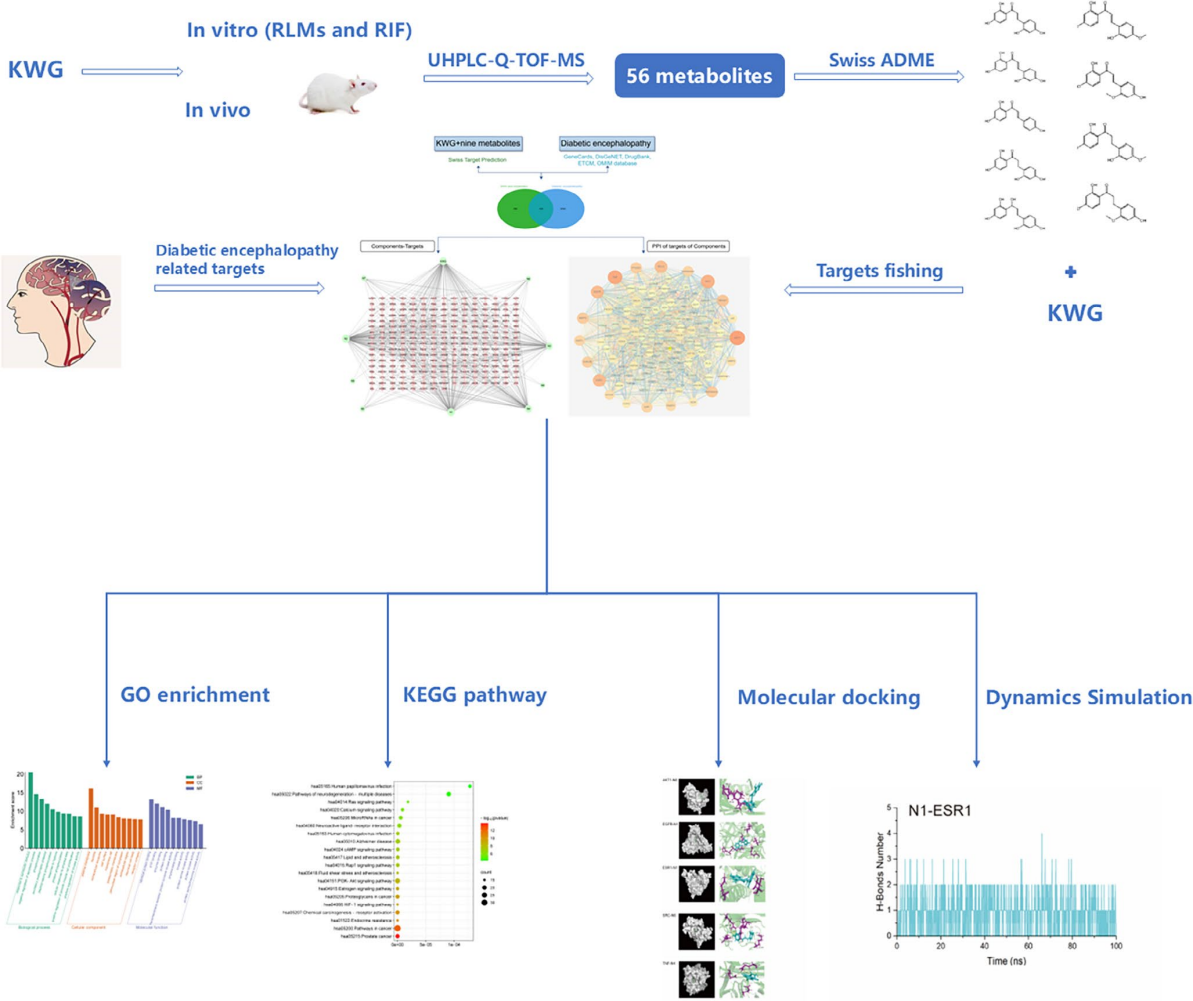
The complete research process.

This study aims to (1) identify KWG metabolites in vivo (blood, urine, bile, and feces) and in vitro (liver microsomes and intestinal bacteria), (2) explore the binding affinities and interactions of KWG and its metabolites with DE‐related targets, and (3) uncover the molecular mechanisms underlying KWG's neuroprotective effects (Wang et al. 2024). The complete research process is illustrated in Figure 2. By integrating experimental and computational methods, we provide a comprehensive understanding of KWG's metabolic activation and its potential as a therapeutic agent for DE.

## 2 Materials and Methods

### 2.1 Chemicals and Reagents

The reference standards (purity > 98%) of KWG (CAS:75629‐19‐5) were purchased from Shanghai Shifeng Biological Technology Co. Ltd. (Chengdu, China). Male rat liver microsomes (SD) stock solution (20 mg/mL) and NADPH generation system [NRS (A) M10001.2022001] were purchased from Wuhan Pratt Biomedical Co. Ltd. Acetonitrile (HPLC grade) and formic acid (Chromatographic pure) were purchased from Thermo Fisher Scientific Company. De‐ionized water was prepared using a Milli‐Q water purification system (Millipore, ELIX100, USA). Carboxymethyl cellulose (CMC‐Na) and other chemicals were analytical grade (Tianjin Chemical Corporation, People's Republic of China). L‐cysteine, L‐ascorbic acid, beef paste, trypsin, and nutrient AGAR were purchased from Beijing Aoboxing Biotechnology Co., LTD.

### 2.2 Instruments and Conditions

Chromatographic separations were performed on a Phenomenex kinetex C18 column (2.1 × 100 mm, 1.7 μm) using a Shimadzu UHPLC system (Kyoto, Japan). The mobile phase was made up of 0.1% aqueous formic acid (A) and acetonitrile (B) with a flow rate of 0.3 mL/min. The column temperature was set at 40°C. The elution gradient was set as follows: 5% B from 0 to 2 min; 5%–95% B from 2 to 25 min; 95% B from 25 to 30 min; and 95%–5% from 30 to 31 min. The injection volume was 2 μL.

Mass spectrometry detection was carried out using a Triple TOF 5600 instrument (AB SCIEX, CA, USA) equipped with a Duo‐Spray ion source. The sample was ionized by electrospray ionization (ESI) and negative ion mode was used for data acquisition. The ion source voltage was −4500 V, the ion source temperature was 550°C, desolvation voltage (DP) was −60 V, the collision energy (CE) was −40 V ± 15 eV, and the gas pressure for the nebulizer (Gas 1, N2), heating gas (Gas 2, N2), and curtain gas (Curtain Gas) were 55 psi, 55 psi, and 35 psi, respectively. The range for parent ion scan was 50–800 Da, and the range for fragment ion scan was 50–1000 Da. IDA mode was used to collect data, and an automated calibration delivery system (calibration delivery system) was used for online mass number correction. The mass spectrometry acquisition duration was 25 min, during which data were collected utilizing Analyst TF 1.6.1 software. Subsequent data processing was performed using Peak View 1.2 and Molecule Profiler 1.3 software. The error range for TOF‐MS mass spectrometry was set at 5.0 ppm, with a minimum peak intensity of 200 cps. Online data from both blank and drug samples were then subjected to analysis according to the predetermined data processing parameters in order to identify potential metabolites. The Clog P value was used to distinguish isomers.

### 2.3 Animals and Drug Administration

Eighteen male Sprague–Dawley rats (Certificate No. SCXK 2024‐0001; weighing 200–250 g; aged 7–8 weeks) were procured from SPF (Beijing) Biotechnology Co. Ltd. The rats were maintained under standard conditions (temperature: 24°C ± 2°C; humidity: 55% ± 5%) and subjected to a 12‐h light/12‐h dark cycle. Following a 7‐day acclimatization period, the rats were randomly assigned to six groups, with three rats per group, including three control groups (blank plasma, blank urine and feces, and blank bile) and three experimental groups (drug plasma, drug urine and feces, and drug bile). All procedures were conducted in accordance with the Guide for the Care and Use of Laboratory Animals published by the National Institutes of Health and approved by the Medical Ethics Committee of the second hospital of Hebei Medical University with the ethical institution approval number 2024‐AE236. Prior to the administration of KWG, the rats were fasted for 12 h. KWG was suspended in a 0.25% sodium carboxymethyl cellulose (CMC‐Na) solution at a concentration of 20 mg/kg for oral administration to the experimental group (Liu et al. 2018). An equivalent volume of CMC‐Na solution without KWG was prepared for the control group.

### 2.4 In Vivo Animal Experiment and Sample Pretreatment

#### 2.4.1 Sample Collection

Plasma samples were obtained via retro‐orbital puncture from the orbital fossa vein using glass capillaries at specified time points: 0.083, 0.167, 0.5, 0.75, 1, 1.5, 2, 3, 5, 8, 12, and 24 h post‐administration (Liao et al. 2015). Blood was collected into heparinized plastic tubes and subsequently centrifuged at 4500 rpm for 10 min. The resulting plasma samples were pooled and stored at −80°C until further analysis. For bile sample collection, rats were anesthetized with a urethane normal saline solution (1.5–2 g/kg) administered via intraperitoneal injection following oral administration. Bile was collected through a bile cannula at intervals of 0–2, 2–4, 4–6, and 6–12 h post‐administration (Wang et al. 2020). The bile samples were sealed with parafilm and stored at −80°C until analysis. To collect urine and feces, rats were housed in metabolic cages prior to administration. Urine and fecal samples were collected over a 72‐h period post‐administration (Zhang et al. 2018) and subsequently combined separately. The feces were air‐dried, and all urine and fecal samples were stored at −80°C.

#### 2.4.2 Sample Pretreatment

Plasma, bile, and urine samples were subjected to vortexing for 3 min following the addition of acetonitrile in a 1:3 (v/v) ratio, followed by centrifugation at 12,000 *g* for 10 min. Fecal samples were treated with acetonitrile in a 1:3 (v/v) ratio using sonication, followed by centrifugation at 12,000 *g* for 10 min. The resulting supernatant was evaporated to dryness, and the residue was reconstituted in 100 μL of acetonitrile for further analysis.

### 2.5 In Vitro Metabolism of KWG

#### 2.5.1 In Vitro Metabolism of KWG by Rat Liver Microsomes

A typical incubation mixture was prepared as described in references (Liao et al. 2018). The mixture consisted of 0.1 mol/L of phosphate buffer (pH 7.4), supplemented with 3.3 mmol/L of MgCl_2_, 1.3 mmol/L of β‐NADPH, 1.0 mg/mL of rat liver microsomes, and 100 μmol/L of KWG, resulting in a final volume of 200 μL. Pre‐incubation was conducted at 37°C for 5 min, after which NADPH was added. Following a 90‐min incubation at 37°C in a metabolic shaker, the reactions were terminated by 600 μL of ice‐cold methanol.

#### 2.5.2 In Vitro Metabolism of KWG by Rat Intestinal Flora

The anaerobic culture medium was prepared as follows: K_2_HPO_4_ (37.5 mL, 0.78%), solution A (37.5 mL, containing 0.47% KH_2_PO_4_, 1.18% NaCl, 1.2% (NH_4_)_2_SO_4_, 0.12% CaCl_2_, and 0.25% MgSO_4_·H_2_O), Na_2_CO_3_ (50 mL, 8%), L‐cysteine (0.5 g), L‐ascorbic acid (2 mL, 25%), eurythrol (1 g), tryptone (1 g), and nutrient agar (1 g) were combined and subsequently diluted with distilled water to a final volume of 1 L. The pH of the resulting solution was then adjusted to 7.5–8.0 using 2 M HCl (Chen et al. 2019; Feng et al. 2018).

Three grams of fresh feces were collected from SD rats and subsequently added to 30 mL of anaerobic culture medium that had been prepared previously. The mixture was then vigorously stirred and filtered through medical gauze to obtain a solution of intestinal flora. KWG (100 μL, 1 mg/mL in DMSO) was added to 1 mL of the intestinal flora solution, and the mixture was transferred into a nitrogen gas bag. Following a 12‐h incubation at 37°C, the reaction was terminated by the addition of 4 mL of methanol.

#### 2.5.3 Sample Pretreatment

The in vitro sample preparation procedure was as follows: the mixture was first vortexed and then centrifuged at 21,380 *g* for a duration of 10 min. Following this, the supernatant was harvested and evaporated to dryness using a stream of nitrogen gas. The resulting residue was subsequently reconstituted in 100 μL of methanol and stored at −20°C until further analysis.

### 2.6 Network Pharmacology, Molecular Docking and Dynamics Simulations

#### 2.6.1 Network Pharmacology

The structures of KWG metabolites were converted into “.sdf” format and subsequently uploaded to Swiss ADME (http://www.swissadme.ch/) for screening based on gastrointestinal absorption and drug‐likeness (Wang et al. 2024). The active metabolite structures were then imported into Swiss Target Prediction (http://www.swisstargetprediction.ch/) to predict potential targets of these active metabolites and KWG. Potential targets associated with DE were identified through searches in several databases, including GeneCards (http://www.genecards.org/), DrugBank (https://www.drugbank.ca), Disgenet (https://www.disgenet.org/), OMIM (https://omim.org/), and ETCM (http://www.tcmip.cn/ETCM/). A Venn diagram was constructed to illustrate the intersection of compounds and DE targets, allowing for the identification of common targets (Bardou et al. 2014). These intersection targets were analyzed using the STRING database and visualized through the Cytoscape 3.7.0 software to construct a protein–protein interaction (PPI) network. Core nodes within the PPI network were identified by evaluating three topological parameters: Betweenness Centrality (BC), Closeness Centrality (CC), and Degree Centrality (DC). The CytoNCA plugin in Cytoscape was employed for the identification of core targets (Xie et al. 2023; Sabarathinam et al. 2024), followed by molecular docking studies of these core targets with KWG and the active metabolites.

#### 2.6.2 Molecular Docking

Molecular docking of KWG, active metabolites, and AGEs inhibitor aminoguanidine with the core target was conducted using AutoDockTools 1.5.7 software (Molecular Graphics Laboratory, The Scripps Research Institute, USA). The reliability of the predictions was assessed based on the lowest conformational binding energy. Subsequently, the molecular docking results were visualized in three dimensions using PyMOL v2.6 software (http://www.pymol.org/pymol) to elucidate the mechanism of action between the target and the active components.

#### 2.6.3 Dynamics Simulations

Molecular dynamics simulations were carried out utilizing the Amber 20 software. For parameterization, the Amber FF19SB force field was applied to proteins, while the Generalized Amber Force Field (GAFF) was employed for ligands. Solvation was achieved using the TIP3P water model, and Na+/Cl− ions were introduced to ensure system charge neutrality. The steepest descent method was used for energy minimization. System heating from 0 K to 298.15 K was conducted under NVT ensemble conditions, followed by equilibration in the NPT ensemble at 298.15 K and 1 bar pressure. A 100 ns molecular dynamics simulation was then executed, with trajectory data recorded for further analysis.

## 3 Results

### 3.1 Mass Cleavage Pattern of KWG

**FIGURE 3:**
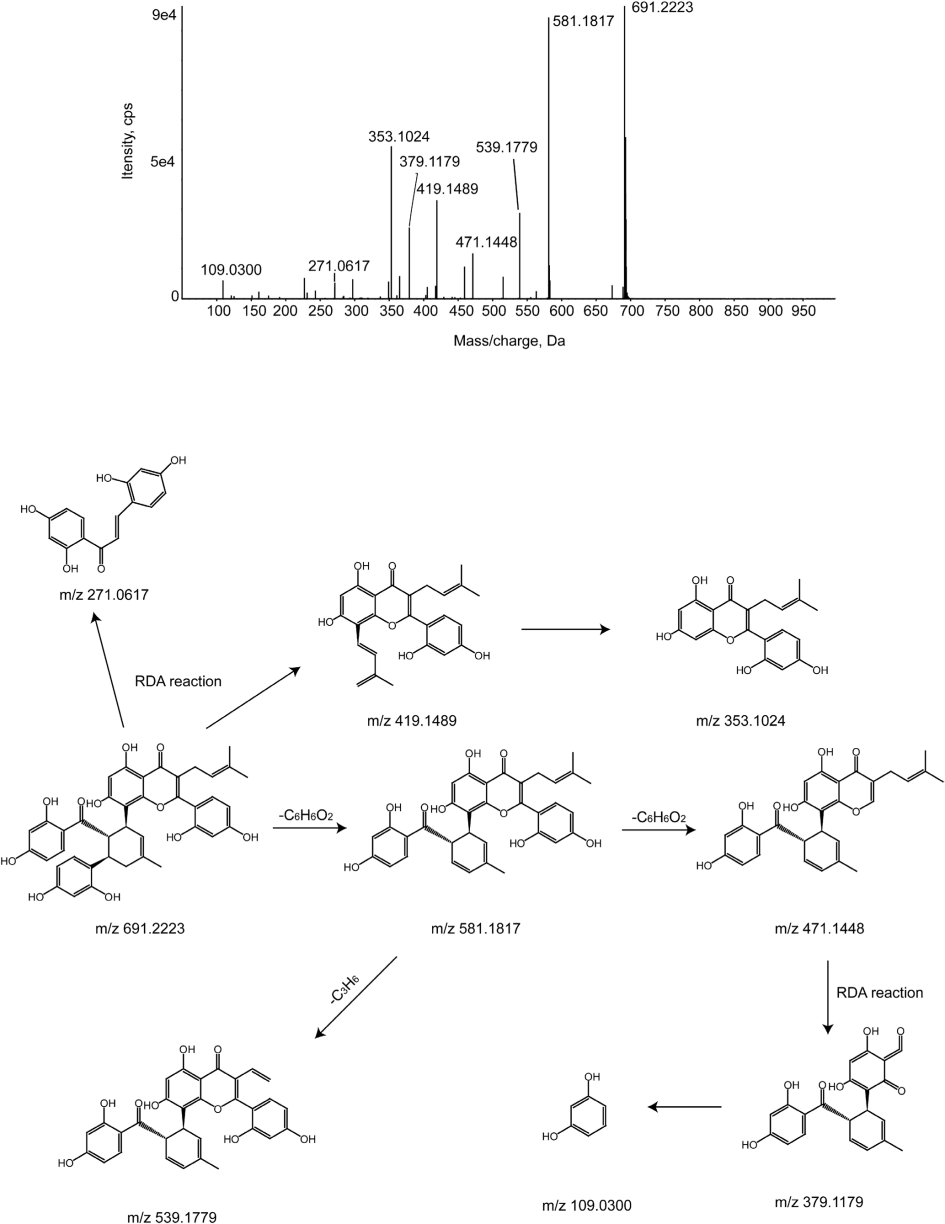
MS^2^ spectrum and fragmentation pathways of KWG.

KWG eluted at 16.76 min with a deprotonated molecular ion [M‐H]^−^ at *m/z* 691.2223, representing the molecular formula C_40_H_36_O_11_ under the experimental conditions. According to the MS/MS mass spectrum of KWG, its characteristic fragment ions were *m/z* 581.1817, 471.1448, 419.1489, 379.1179, 353.1024, 271.0617, and 109.0300. The product ions at *m/z* 581.1817 and 471.1448 were formed by subsequently losses of C_6_H_6_O_2_. The product ions at *m/z* 539.1779 were observed by the elimination of loss of C_3_H_6_ on the basis of *m/z* 581.1817. The product ions at *m/z* 379.1179 were found as a result of the Retro‐Diels–Alder reaction (RDA reaction) on the basis of *m/z* 471.1448. Furthermore, the RDA reaction of the prototype resulted in the generation of fragment ions at *m/z* 419.1489 and 271.0617 (Luo et al. 2021); the product ion at *m/z* 353.1024 was obtained by the loss of C_5_H_8_ on the basis of the product ion at *m/z* 419.1489. The MS^2^ spectrum and cleavage pattern of KWG were shown in Figure 3.

### 3.2 Characterizing the Metabolites of KWG in Rats

**FIGURE 4:**
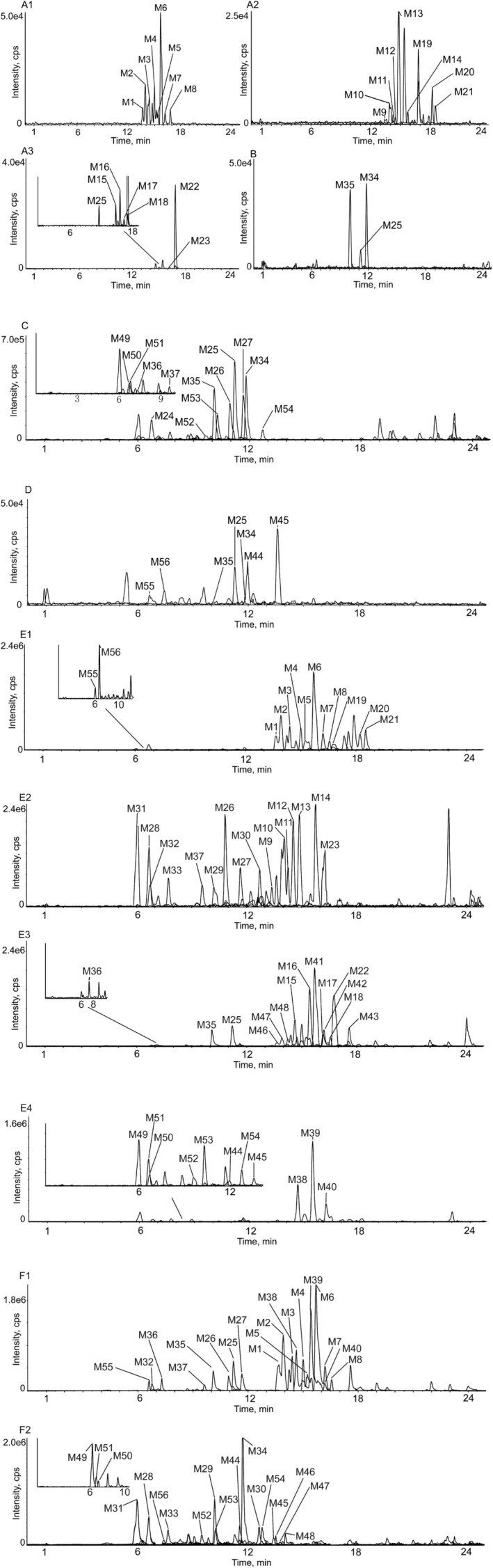
XICs of all the KWG metabolites in vivo and in vitro (A, B, C, D, E, F) (A, 1–3 in liver microsomes, B in urine, C in blood, D in bile, E in feces, F in intestinal flora).

**FIGURE 5:**
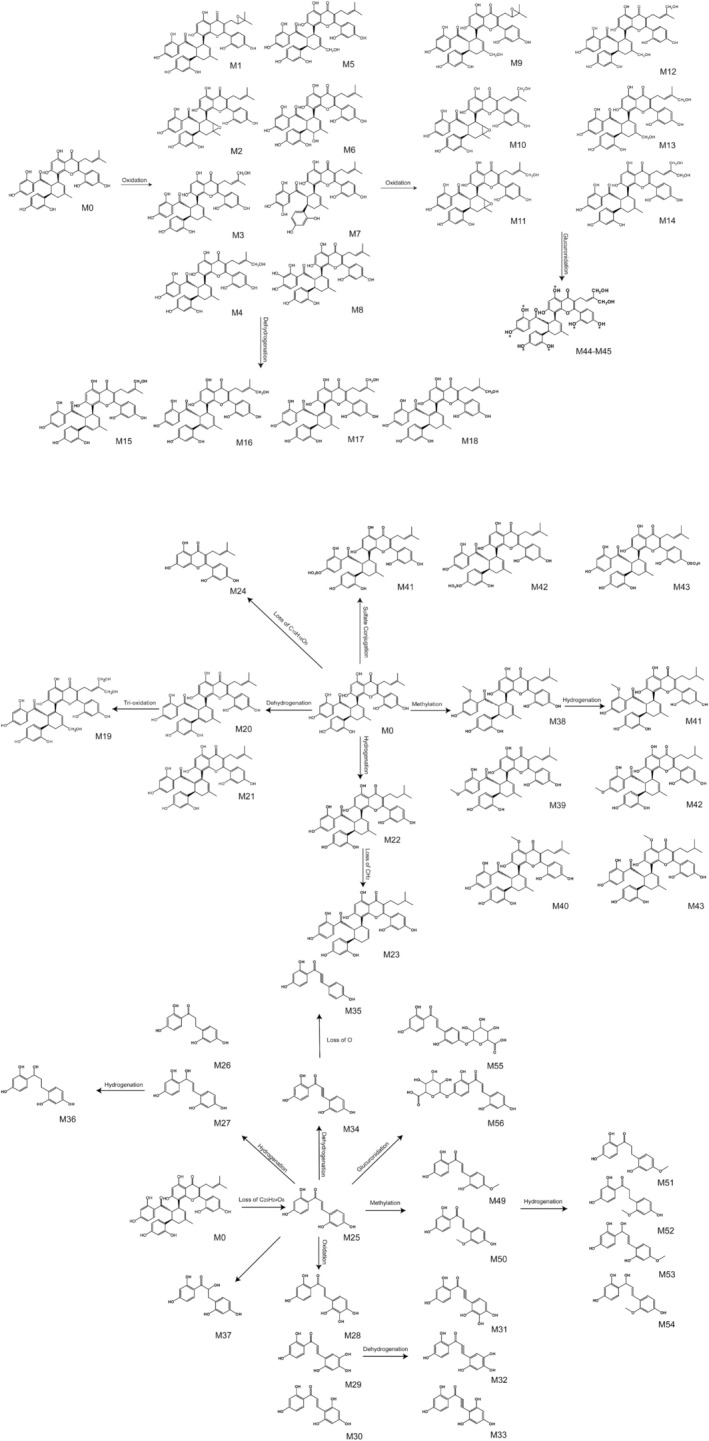
Metabolic profile and proposed metabolic pathways of KWG in vivo and in vitro (*—possible site of metabolites).

Utilizing UHPLC‐Q‐TOF‐MS technology, a thorough analysis was performed, resulting in the identification of 56 metabolites of KWG, which included 37 phase I metabolites and 19 phase II metabolites, excluding the parent compound. The extracted ion chromatogram (XIC) of KWG metabolites is illustrated in Figures 4 and 5 presents the structural elucidation of these metabolites, emphasizing the chemical modifications that occurred during the metabolic process. Detailed information regarding the identified metabolites is provided in Table 1.

**TABLE 1:** Summary of metabolites of KWG detected in vivo and in vitro.

Compound ID	Formula	Measured mass ([M‐H]^−^)	Calculated mass ([M‐H]^−^)	Error (ppm)	*t*_*R*_ (min)	MS/MS fragments	Composition shift	RLMs	RIF	Urine	Feces	Bile	Blood
M1	C_40_H_36_O_12_	707.2134	707.2135	0.1	13.64	597.1790, 487.1425, 435.1450, 353.1036, 271.0633, 109.0308	Oxidation	+	+	−	+	−	−
M2	C_40_H_36_O_12_	707.2135	707.2137	0.2	13.92	597.1770, 487.1427, 435.1447, 353.1032, 271.0632, 109.0301	Oxidation	+	+	−	+	−	−
M3	C_40_H_36_O_12_	707.2131	707.2130	−0.1	14.40	597.1787, 487.1427, 435.1463, 353.1040, 271.0630, 109.0305	Oxidation	+	+	−	+	−	−
M4	C_40_H_36_O_12_	707.2129	707.2131	0.5	15.00	597.1789, 487.1426, 435.1467, 353.1043, 271.0637, 109.0303	Oxidation	+	+	−	+	−	−
M5	C_40_H_36_O_12_	707.2126	707.2124	−1.9	15.32	597.1791, 487.1432, 435.1466, 353.1049, 271.0632, 109.0309	Oxidation	+	+	−	+	−	−
M6	C_40_H_36_O_12_	707.2123	707.2120	−0.8	15.71	597.1773, 487.1421, 435.1456, 353.1030, 271.0628, 109.0303	Oxidation	+	+	−	+	−	−
M7	C_40_H_36_O_12_	707.2136	707.2138	0.3	16.21	597.1771, 487.1420, 419.1452, 353.1031, 287.0629, 109.0306	Oxidation	+	+	−	+	−	−
M8	C_40_H_36_O_12_	707.2132	707.2137	2.2	16.55	597.1778, 487.1422, 419.1450, 353.1030, 287.0630, 109.0309	Oxidation	+	+	−	+	−	−
M9	C_40_H_36_O_13_	723.2083	723.2085	0.3	13.37	613.1747, 487.3136, 451.1422, 353.1034, 271.0610, 109.0309	Di‐oxidation	+	−	−	+	−	−
M10	C_40_H_36_O_13_	723.2082	723.2082	0.1	14.01	613.1749, 487.3130, 451.1421, 353.1036, 271.0615, 109.0302	Di‐oxidation	+	−	−	+	−	−
M11	C_40_H_36_O_13_	723.2108	723.2110	3.2	14.26	613.1744, 487.3131, 451.1410, 353.1038, 271.0627, 109.0301	Di‐oxidation	+	−	−	+	−	−
M12	C_40_H_36_O_13_	723.2091	723.2090	−0.1	14.54	613.1747, 487.3130, 451.1416, 353.1031, 271.0622, 109.0304	Di‐oxidation	+	−	−	+	−	−
M13	C_40_H_36_O_13_	723.2099	723.2102	1.1	14.88	613.1745, 487.3133, 451.1415, 353.1036, 271.0624, 109.0306	Di‐oxidation	+	−	−	+	−	−
M14	C_40_H_36_O_13_	723.2098	723.2100	0.3	15.76	613.1748, 487.3132, 451.1417, 353.1034, 271.0623, 109.0307	Di‐oxidation	+	−	−	+	−	−
M15	C_40_H_34_O_12_	705.1977	705.2000	1.2	14.62	595.1631, 485.1262, 433.1293, 353.1033, 271.0622, 109.0303	Oxidation; Dehydrogenation	+	−	−	+	−	−
M16	C_40_H_34_O_12_	705.1999	705.2002	0.3	15.43	595.1645, 485.1266, 433.1300, 353.1032, 271.0625, 109.0308	Oxidation; Dehydrogenation	+	−	−	+	−	−
M17	C_40_H_34_O_12_	705.2001	705.2000	−0.1	16.16	595.1639, 485.1259, 433.1304, 353.1036, 271.0630, 109.0304	Oxidation; Dehydrogenation	+	−	−	+	−	−
M18	C_40_H_34_O_12_	705.2009	705.2010	0.6	16.43	595.1641, 485.1260, 433.1302, 353.1033, 271.0631, 109.0307	Oxidation; Dehydrogenation	+	−	−	+	−	−
M19	C_40_H_34_O_14_	737.1875	737.1890	1.9	16.76	719.2365, 701.1377, 683.1299, 581.1866, 471.1491, 417.1530, 351.1032, 271.0635, 109.0310	Dehydrogenation; Tri‐oxidation	+	−	−	+	−	−
M20	C_40_H_34_O_11_	689.2028	689.2075	0.5	18.22	579.1707, 469.1311, 417.1352, 351.1029, 271.0628, 109.0303	Dehydrogenation	+	−	−	+	−	−
M21	C_40_H_34_O_11_	689.2027	689.2060	0.4	18.53	579.1708, 469.1313, 417.1350, 351.1031, 271.0623, 109.0307	Dehydrogenation	+	−	−	+	−	−
M22	C_40_H_38_O_11_	693.2341	693.2326	−2.1	16.74	583.1904, 473.1539, 421.1577, 355.1031, 271.0623, 109.0306	Hydrogenation	+	−	−	+	−	−
M23	C_39_H_36_O_11_	679.2184	679.2188	1.0	16.24	569.1484, 459.1100, 407.1157, 353.1086, 271.0624, 109.0303	Hydrogenation; Loss of CH_2_	+	−	−	+	−	−
M24	C_20_H_18_O_6_	353.1030	353.1011	−3.1	6.73	271.0624, 109.0302	Loss of C_10_H_18_O_5_	−	−	−	+	−	+
M25	C_15_H_12_O_5_	271.0612	271.0620	2.2	11.21	165.0196, 151.0047, 137.0253, 109.0308	Loss of C_25_H_24_O_6_	+	+	+	+	+	+
M26	C_15_H_14_O_5_	273.0768	273.0773	1.9	10.95	167.0362, 151.0044, 137.0239, 109.0308	Loss of C_25_H_24_O_6_; Hydrogenation	−	+	−	+	−	+
M27	C_15_H_14_O_5_	273.0770	273.0772	1.0	11.67	167.0364, 151.0045, 137.0236, 109.0301	Loss of C_25_H_24_O_6_; Hydrogenation	−	+	−	+	−	+
M28	C_15_H_12_O_6_	287.0561	287.0568	0.7	6.67	269.1202, 165.0198, 151.0048, 137.0247, 109.0309	Loss of C_25_H_24_O_6_; Oxidation	−	+	−	+	−	−
M29	C_15_H_12_O_6_	287.0565	287.0570	1.0	10.28	269.1201, 165.0197, 151.0045, 137.0249, 109.0302	Loss of C_25_H_24_O_6_; Oxidation	−	+	−	+	−	−
M30	C_15_H_12_O_6_	287.0569	287.0561	−0.9	12.71	269.1205, 165.0193, 151.0041, 137.0243, 109.0302	Loss of C_25_H_24_O_6_; Oxidation	−	+	−	+	−	−
M31	C_15_H_10_O_6_	285.0406	285.0410	0.4	6.04	267.1190, 163.0301, 151.0049, 137.0246, 109.0300	Loss of C_25_H_24_O_6_; Oxidation; Dehydrogenation	−	+	−	+	−	−
M32	C_15_H_10_O_6_	285.0409	285.0420	2.0	6.73	267.1196, 163.0309, 151.0043, 137.0247, 109.0301	Loss of C_25_H_24_O_6_; Oxidation; Dehydrogenation	−	+	−	+	−	−
M33	C_15_H_10_O_6_	285.0401	285.0423	2.9	7.73	267.1191, 163.0304, 151.0043, 137.0244, 109.0307	Loss of C_25_H_24_O_6_; Oxidation; Dehydrogenation	−	+	−	+	−	−
M34	C_15_H_10_O_5_	269.0455	269.0462	2.7	11.82	163.0291, 151.0050, 137.0221, 109.0310	Loss of C_25_H_24_O_6_; Dehydrogenation	−	+	+	+	+	+
M35	C_15_H_10_O_4_	253.0563	253.0518	−1.8	10.11	160.0177, 151.0041, 137.0301, 109.0309	Loss of C_25_H_24_O_6_; Dehydrogenation; Loss of O	−	+	+	+	+	+
M36	C_15_H_16_O_5_	275.0925	275.0989	1.5	7.32	166.0645, 151.0039, 137.0301, 109.0310	Loss of C_25_H_24_O_6_; Di‐Hydrogenation	−	+	−	+	−	+
M37	C_15_H_14_O_6_	289.0717	289.0739	3.1	9.63	179.0357, 163.0410, 151.0041, 137.0222, 109.0300	Loss of C_25_H_24_O_6_; Internal Hydrolysis	−	+	−	+	−	+
M38	C_41_H_38_O_11_	705.2341	705.2342	0.3	14.62	596.1629, 485.1262, 419.1451, 285.0730, 353.1039, 109.0303	Methylation	−	+	−	+	−	−
M39	C_41_H_38_O_11_	705.2339	705.2328	−2.1	15.43	596.1630, 485.1260, 419.1453, 285.0736, 353.1033, 109.0308	Methylation	−	+	−	+	−	−
M40	C_41_H_38_O_11_	705.2345	705.2349	0.7	16.16	596.1633, 485.1260, 433.1289, 271.0627, 353.1038, 109.0302	Methylation	−	+	−	+	−	−
M41	C_41_H_40_O_11_	707.2497	707.2450	0.4	15.70	597.1733, 487.1421, 421.1080, 353.1030, 285.0435, 109.0303	Hydrogenation; Methylation	−	−	−	+	−	−
M42	C_41_H_40_O_11_	707.2500	707.2454	0.9	16.20	597.1739, 487.1427, 421.1086, 353.1033, 285.0439, 109.0308	Hydrogenation; Methylation	−	−	−	+	−	−
M43	C_41_H_40_O_11_	707.2505	707.2444	−1.6	17.58	597.1740, 487.1426, 435.1088, 353.1039, 271.0629, 109.0305	Hydrogenation; Methylation	−	−	−	+	−	−
M44	C_46_H_46_O_18_	885.2611	885.2619	0.8	11.92	775.2762, 665.1444, 451.1673, 353.1080, 109.0309	Di‐oxidation; Glucuronidation	−	+	−	+	+	−
M45	C_46_H_46_O_18_	885.2620	885.2638	1.5	13.51	775.2767, 665.1442, 451.1676, 353.1082, 109.0306	Di‐oxidation; Glucuronidation	−	+	−	+	+	−
M46	C_40_H_37_O_14_S	772.1831	772.1842	2.0	13.61	691.2270, 581.1813, 471.1527, 353.1100, 109.0305	Sulfate Conjugation	−	+	−	+	−	−
M47	C_40_H_37_O_14_S	772.1836	772.1830	−1.2	13.86	691.2276, 581.1814, 471.1523, 353.1106, 109.0307	Sulfate Conjugation	−	+	−	+	−	−
M48	C_40_H_37_O_14_S	772.1830	772.1844	1.9	14.15	691.2271, 581.1816, 471.1529, 353.1104, 109.0309	Sulfate Conjugation	−	+	−	+	−	−
M49	C_16_H_14_O_5_	285.0768	285.0738	−2.9	6.04	271.0621, 165.0198, 151.0051, 137.0231, 109.0297	Loss of C_25_H_24_O_6_; Methylation	−	+	−	+	−	+
M50	C_16_H_14_O_5_	285.0799	285.0891	2.3	6.73	271.0629, 165.0193, 151.0041, 137.0230, 109.0300	Loss of C_25_H_24_O_6_; Methylation	−	+	−	+	−	+
M51	C_16_H_16_O_5_	287.0925	287.0930	0.6	6.67	257.1188, 239.1095, 167.0397, 151.0048, 137.0267, 109.0302	Loss of C_25_H_24_O_6_; Methylation; Hydrogenation	−	+	−	+	−	+
M52	C_16_H_16_O_5_	287.0931	287.0939	0.8	9.61	257.1182, 239.1094, 167.0392, 151.0041, 137.0262, 109.0306	Loss of C_25_H_24_O_6_; Methylation; Hydrogenation	−	+	−	+	−	+
M53	C_16_H_16_O_5_	287.0921	287.0941	2.1	10.28	257.1180, 239.1091, 167.0392, 151.0043, 137.0261, 109.0306	Loss of C_25_H_24_O_6_; Methylation; Hydrogenation	−	+	−	+	−	+
M54	C_16_H_16_O_5_	287.0933	287.0921	−1.2	12.71	257.1179, 239.1091, 167.0390, 151.0041, 137.0263, 109.0309	Loss of C_25_H_24_O_6_; Methylation; Hydrogenation	−	+	−	+	−	+
M55	C_21_H_20_O_11_	447.0932	447.0921	−1.0	6.05	271.0601, 165.0190, 151.0048, 137.0249, 109.0302	Loss of C_25_H_24_O_6_; Glucuronidation	−	+	−	+	+	−
M56	C_21_H_20_O_11_	447.0929	447.0926	0.2	6.73	271.0621, 165.0194, 151.0047, 137.0253, 109.0306	Loss of C_25_H_24_O_6_; Glucuronidation	−	+	−	+	+	−

*Note:* +: Detected; −: Undetected.

### 3.3 Identification of Phase I Metabolites

#### 3.3.1 Oxidation. Metabolites M1–M8

M1–M8 represented the deprotonated molecular ion [M‐H]^−^ were observed at *m/z* 707.2135, 707.2137, 707.2130, 707.2131, 707.2124, 707.2120, 707.2138, 707.2137, which were 16 Da larger than KWG, indicating that the M1‐M8 might be monooxidized KWG. In the M1‐M8 MS^2^ spectra, two prominent ions at *m/z* 597 and 487 were identified by successive loss of C_6_H_6_O_2_, which indicated oxidation reaction did not occur on the B ring and B′ ring. The M1–M6 MS^2^ spectra revealed the presence of characteristic ions at *m/z* 435 (*m/z* 419 + 16 Da), indicating potential oxidation reactions occurring on the A ring, C′ ring, or isopentenyl moiety. In the M7–M8 MS^2^ spectra, the fragment ion at *m/z* 287 (*m/z* 271 + 16 Da) was formed because of the RDA reaction, confirming that the A' ring contained the added O atom. Moreover, the Clog P values of M1–M8 were 4.24811, 4.45142, 4.53122, 4.57890, 4.65783, 4.78931, 5.12543, and 5.24811, respectively. So, M1–M8 were characterized based on this fact.

#### 3.3.2 Di‐Oxidation. Metabolites M9–M14

M9–M14 presented [M‐H]^−^ at *m/z* 723 with the formula of C_40_H_36_O_13_, classifying into di‐oxidation metabolite. These metabolites showed a mass 32 Da greater than that of KWG. They could produce fragment ions at *m/z* 613 [M‐H‐ C_6_H_6_O_2_]^−^, 487[M‐H‐2C_6_H_6_O_2_]^−^. The representative ions at *m/z* 451 (*m/z* 419 + 32 Da, RDA), 353, 271, 109 appeared in the M9–M14 MS^2^ spectrum. Their Clog P values were calculated to be 3.28743, 3.41233, 3.42365, 3.46784, 3.51231, 3.54376.

#### 3.3.3 Oxidation + Dehydrogenation. Metabolites M15–M18

M15–M18 all exhibited deprotonated ions with *m/z* values of 705.2000, 705.2002, 705.2000, and 705.2010, respectively, representing a decrease in mass by 2 Da compared to M1–M8. The precursor ion of the M15–M18 cleaved into ions of *m/z* 595 (loss of C_6_H_6_O_2_ from *m/z* 705), 149 (loss of C_6_H_6_O_2_ from *m/z* 595); the typical fragment ion of RDA reaction at *m/z* 433 (419 + 14 Da) implied that the oxidation occurred at the isopentenyl group. They were deduced as C_40_H_34_O_12_, and the Clog P values of M15–M18 were 4.06325, 4.067390, 4.190324, and 4.28784, respectively.

#### 3.3.4 Tri‐Oxidation + Dehydrogenation. Metabolites M19

The retention time of M19 (C_40_H_34_O_14_) was 16.76 min, with a detection at *m/z* 737.1890, indicating a mass increase of 32 Da compared to M15–M18, thus confirming the occurrence of a tri‐oxidation and dehydrogenation reaction. Some representative ions at *m/z* 581.1866, 471.1491, 353.1032, 271.0635, 109.0310 appeared in the MS^2^ spectrum.

#### 3.3.5 Dehydrogenation. Metabolites M20–M21

Metabolites M20 and M21 (C_40_H_34_O_11_) eluting at 18.22 and 18.53 min were found in the extracted ion chromatograms of *m/z* 689.2075 and 689.2060, respectively. The product ions at *m/z* 579, 469, 417, 351 were 2 Da higher than 581, 471, 419, 353 of KWG. The Clog P values were calculated to be 5.12123 and 5.20571, leading to the recognition of M20 and M21.

#### 3.3.6 Hydrogenation Metabolites M22

Metabolite M22 (C_40_H_38_O_11_) eluted at a retention time of 16.74 min on the UPLC system. Its deprotonated molecular ion [M‐H]^−^ at *m/z* 693.2326 showed an addition of two hydrogen atoms compared to KWG. The peaks at *m/z* 583.1904, 473.1539, 421.1577, and 355.1031 exhibited a mass increase of 2 Da compared to the corresponding peaks at *m/z* 581.1817, 471.1448, 419.1489, and 353.1024 for KWG, indicating a hydrogenation reaction occurred in this region.

#### 3.3.7 Hydrogenation + Loss of CH_2_ Metabolites M23

M23 was detected at *m/z* 679.2188, with a mass 12 Da smaller than that of KWG. Furthermore, M23 exhibited a single elution time at 16.24 min and had the molecular formula C_39_H_36_O_11_. According to the speculation of Molecule Profiler 1.3 software, KWG underwent loss of CH_2_ and hydrogenation, and then produced M23. Additionally, several major ions at *m/z* 569.1484, 459.1100, and 407.1157 were observed, indicating the loss of a CH_2_ group from KWG and simultaneous isopentenyl reduction.

#### 3.3.8 Loss of C_10_H_18_O_5_ Metabolites M24

Metabolite M24 (C_20_H_18_O_6_) exhibited a deprotonated molecule ion [M‐H]^−^ at *m/z* 353.1011, which closely resembled the fragment ion at *m/z* 353.1024 of the parent drug KWG. Characteristic fragment ions at *m/z* 271.0624 were observed as a result of the RDA reaction.

#### 3.3.9 Loss of C_25_H_24_O_6_ Metabolites M25

Metabolite M25 exhibited [M‐H]^−^ at *m/z* 271.0620 with the molecular formula C_15_H_12_O_5_. Fragment ions of M25 were observed at *m/z* 165.0196 [M‐H‐ C_6_H_2_O_2_]^−^, 151.0047 [M‐H‐ C_6_H_2_O_2_‐ CH_2_]^−^, and 137.0253 [M‐H‐ C_6_H_2_O_2_‐ 2CH_2_]^−^. The characteristic ion at *m/z* 109.0308 was identified in the MS^2^ spectrum of M25. Based on the structure and molecular formula, it is speculated that M25 is 2,2′,4,4′‐tetrahydroxychalcone.

#### 3.3.10 Loss of C_25_H_24_O_6_ + Hydrogenation Metabolites M26–M27

Metabolites M26 and M27 (C_15_H_14_O_5_) were observed as deprotonated molecules [M‐H]^−^ at *m/z* 273.0773 and 273.0772. The mass increase of 2 Da compared to M25 suggested the addition of two hydrogen atoms to M25. The identical fragment ion at *m/z* 167 was found to be 2 Da higher than the fragment ion at *m/z* 165 of M25, while the fragment ions at *m/z* 151,137, and109 were consistent with those from M25 (*m/z* 151.0047, 137.0253, and 109.0308). Additionally, the Clog P values were determined to be 2.05672 and 2.13267 for M26 and M27.

#### 3.3.11 Loss of C_25_H_24_O_6_ + Oxidation Metabolites M28–M30

Metabolites M28–M30 (C_15_H_12_O_6_) were eluted at 6.67, 10.28, and 12.71 min with [M‐H]^−^ at *m/z* 287.0568, 287.0570, and 287.0561, showing an increase of 16 Da compared with M25. The characteristic product ions of M28–M30 were observed at *m/z* 165, 151, 137, and 109, consistent with those from M25, indicating oxidation occurred at the B' ring. The Clog P values for M28–M30 were determined to be 1.64580, 1.64678, and 1.64983, respectively.

#### 3.3.12 Loss of C_25_H_24_O_6_ + Oxidation + Dehydrogenation Metabolites M31–M33

Metabolites M31–M33 (C_15_H_10_O_6_) were detected with quasi‐molecular ions at *m/z* 285.0410, 285.0420, and 285.0423, respectively, which were observed to be lower by 2 Da compared to those of M28–M30, indicating a dehydrogenation reaction had occurred. The product ion at *m/z* 267 and *m/z* 163 was found to be reduced by 2 Da compared to the values for M28–M30 (*m/z* 269 and *m/z* 165). Additionally, the fragment ion at *m/z* 137 and *m/z* 109 was consistent with that from M28–M30. Furthermore, the Clog P values were determined as 1.56287 for M31, 1.56378 for M32, and 1.56495 for M33.

#### 3.3.13 Loss of C_25_H_24_O_6_ + Dehydrogenation Metabolites M34

Metabolite M34 (C_15_H_10_O_5_) was eluted at a retention time of 11.82 min on the UPLC system. Its deprotonated molecular ion [M‐H]^−^ at *m/z* 269.0462 exhibited a reduction of two hydrogen atoms compared to M25. The fragment ions at *m/z* 163.0291 were 2 Da less than the 165.0196 of M25, indicating a loss of H_2_ reaction located in M25. Meanwhile, the characteristic fragment ions at *m/z* 151.0050, 137.0221, and 109.0310 were consistent with those of M25.

#### 3.3.14 Loss of C_25_H_24_O_6_ + Dehydrogenation + Loss of O Metabolites M35

Metabolite M35 (C_15_H_10_O_4_) exhibited a UHPLC profile with a retention time of 10.11 min, revealing quasi‐molecular ions [M‐H]^−^ at *m/z* 253.0518, which is 16 Da less than that of M34. It generated a characteristic fragment ion at *m/z* 160.0177 due to the loss of C_6_H_5_O, indicating the absence of oxygen in M34. Additionally, the ions observed at *m/z* 151.0041, 137.0301, and 109.0309 were consistent with those found in M34.

#### 3.3.15 Loss of C_25_H_24_O_6_ + Di‐Hydrogenation Metabolites M36

M36 (C_15_H_16_O_5_) yielded the deprotonated molecule ion [M‐H]‐ at *m/z* 275.0989, which was 4 Da higher than that of M25. It generated a distinctive fragment ion at 166.0645 by losing C_6_H_5_O_2_. The diagnostic product ions at *m/z* 151.0039, 137.0301, and 109.0310 were observed in the MS/MS spectrum of M36. Based on this evidence, M36 was identified.

#### 3.3.16 Loss of C_25_H_24_O_6_ + Internal Hydrolysis Metabolites M37

Metabolites M37 (C_15_H_14_O_6_) exhibited quasi‐molecular ions at *m/z* 289.0739 and were eluted at 9.36 min in the MS^2^ spectrum, which was 18 Da higher than that of M25. Additionally, the typical fragment ions at *m/z* 151.0042, 137.0222, and 109.0300 closely matched those from M25 (*m/z* 151.0047, 137.0253, and 109.0308), suggesting that M37 underwent an addition of one H_2_O compared to M25.

### 3.4 Identification of Phase II Metabolites

#### 3.4.1 Methylation Metabolites M38–M40

The metabolites M38–M40 were eluted at retention times of 14.62 min, 15.43 min, and 16.16 min, with deprotonated molecule ions [M‐H]^−^ observed at *m/z* 705.2342, 705.2328, and 705.2349, respectively. The mass increment of 14 Da compared to KWG suggested the addition of a methyl group to the parent compound. In M38–M40, the key fragment ions at *m/z* 595 and 485 were 14 Da higher than 581 and 471 from KWG, respectively, implying that a methylation reaction did not occur in the B ring and B′ ring. The characteristic ions at *m/z* 285 (*m/z* 271 + 14 Da) were detected in the M38 and M39 MS^2^ spectra, suggesting that methylation took place on the hydroxyl group of the A' ring. In M40, the typical fragment ions at *m/z* 433.1289 (*m/z* 419.1489 + 14 Da)indicated that M40 added one methyl group on the A ring. The position of the methylation reaction of M38–M40 depends on the Clog P values, which were 5.86023, 5.861128, and 5.86875, respectively.

#### 3.4.2 Methylation + Hydrogenation Metabolites M41–M43

Metabolites M41, M42, and M43 (C_41_H_40_O_11_) were identified as isomeric metabolites with deprotonated ions [M‐H]‐ at *m/z* 707, indicating a mass increase of 2 Da compared to M38–M40. Notably, in the MS/MS spectra of M41 and M42, the representative product ions showed *m/z* 421.1080 (*m/z* 419.1489 + 2 Da) and 285.0435 (*m/z* 271.0617 + 14 Da), which were gained by RDA reaction, implying that methylation took place in the hydroxyl group on the A' ring, and hydrogenation occurred in the isopentenyl part. In contrast to this pattern seen in M41–M42 fragmentation patterns, the diagnostic fragment ion at *m/z* 435.1088 was observed for M43 which was higher by 16 Da (CH_2_ + H_2_) than the *m/z* 419.1489 of KWG, indicating methylation took place on A ring's hydroxyl group and H_2_ added to its isopentenyl part. The calculated Clog P values for these compounds were determined as 6.45704, 6.61704, and 6.83495, respectively, resulting in their successful identification.

#### 3.4.3 Di‐ Oxidation + Glucuronidation Metabolites M44–M45

Metabolites M44 and M45 (C_46_H_46_O_18_) were the isomeric metabolites with the deprotonated molecular ions [M‐H]^−^ at *m/z* 885.2619 and 885.2638. They had the characteristic fragment ions at *m/z* 451 (*m/z* 419 + O + O), which were gained through the RDA reaction, suggesting that di‐oxidation occurred on the isopentenyl part. However, the exact glucuronic acid binding site was not certain in this study.

#### 3.4.4 Sulfate Conjugation Metabolites M46–M48

Metabolites M46–M48 exhibited the deprotonated molecule ions [M‐H]^−^ at *m/z* 772.1842, 772.1830, and 772.1844. They owed the characteristic fragment ions at *m/z* 691 [M‐H‐SO_3_H]^−^ under the mass spectrometry conditions, which were in agreement with KWG, implying that sulfate conjugation occurred on KWG. Moreover, the typical fragment ions at *m/z* 581, 471, 353, and 109 were in close agreement with those from KWG. Additionally, typical fragment ions at *m/z* 581, 471, 353, and 109 closely matched those of KWG. The position of sulfate conjugation for M46–M48 correlated with their respective Clog P values of 1.95012, 1.95341, and 1.95768. Then M46, M47, and M48 were identified.

#### 3.4.5 Loss of C_25_H_24_O_6_ + Methylation Metabolites M49–M50

Metabolite M49–M50 (C_16_H_14_O_5_) exhibited a UPLC profile with retention times of 6.04 and 6.73 min, respectively. In the MS/MS spectrum, an [M‐H]^−^ ion at *m/z* 285.0738 and 285.0891 was observed, corresponding to a mass increase of 14 Da compared to M25. Sequential characteristic product ions at *m/z* 271, 165, 151, and 137 were generated through successive loss of CH_2_, C_6_H_2_O_2_, CH_2_, and CH_2_ groups. Additionally, the presence of typical fragment ions at *m/z* 109 closely matched those from M25, suggesting that M49–M50 had added one methyl group relative to M25. Furthermore, the Clog P values for M49–M50 were determined as being 2.29086 and 2.29891, respectively.

#### 3.4.6 Loss of C_25_H_24_O_6_ + Methylation + Hydrogenation Metabolites M51–M54

Metabolites M51–M54 (C_16_H_16_O_5_) were isomeric metabolites with deprotonated ions [M‐H]^−^ at *m/z* 287, indicating a mass increase of 2 Da compared to M49–M50. They exhibited characteristic fragment ions at *m/z* 257 [M‐H‐O‐CH_2_]^−^ and *m/z* 239 [M‐H‐O‐CH_2_‐H_2_O]^−^ under the specified mass spectrometry conditions. The product ion at *m/z*167 was observed to be higher by 2 Da than that of M49–M50; additionally, the presence of identical ions at *m/z* 151, 137, and 109 in comparison with M49–M50 suggested hydrogenation in M49–M50 had occurred. In addition, the Clog P values of M51–M54 were 2.31648, 2.31892, 2.39648, and 2.39987 respectively.

#### 3.4.7 Loss of C_25_H_24_O_6_ + Glucuronidation Metabolites M55–M56

Metabolites M55 and M56 (C_21_H_20_O_11_) were eluted at 6.05 and 6.73 min, exhibiting deprotonated molecular ions at *m/z* 447.0921 and 447.0926, respectively. The fragment ion at *m/z* 271 was generated through the neutral loss of 176 Da, indicating the presence of a single glucuronic acid moiety in M55 and M56. Predominant fragment ions at *m/z* 165, 151, 137, and 109 were consistent with those of M25. Furthermore, the Clog P values for M55–M56 were determined to be 0.16658 and 0.18281, respectively. It is noteworthy that steric hindrance significantly influences the site of reaction occurrence, which necessitated a thorough elucidation of their structures.

### 3.5 Results of Network Pharmacology and Molecular Docking

**FIGURE 6:**
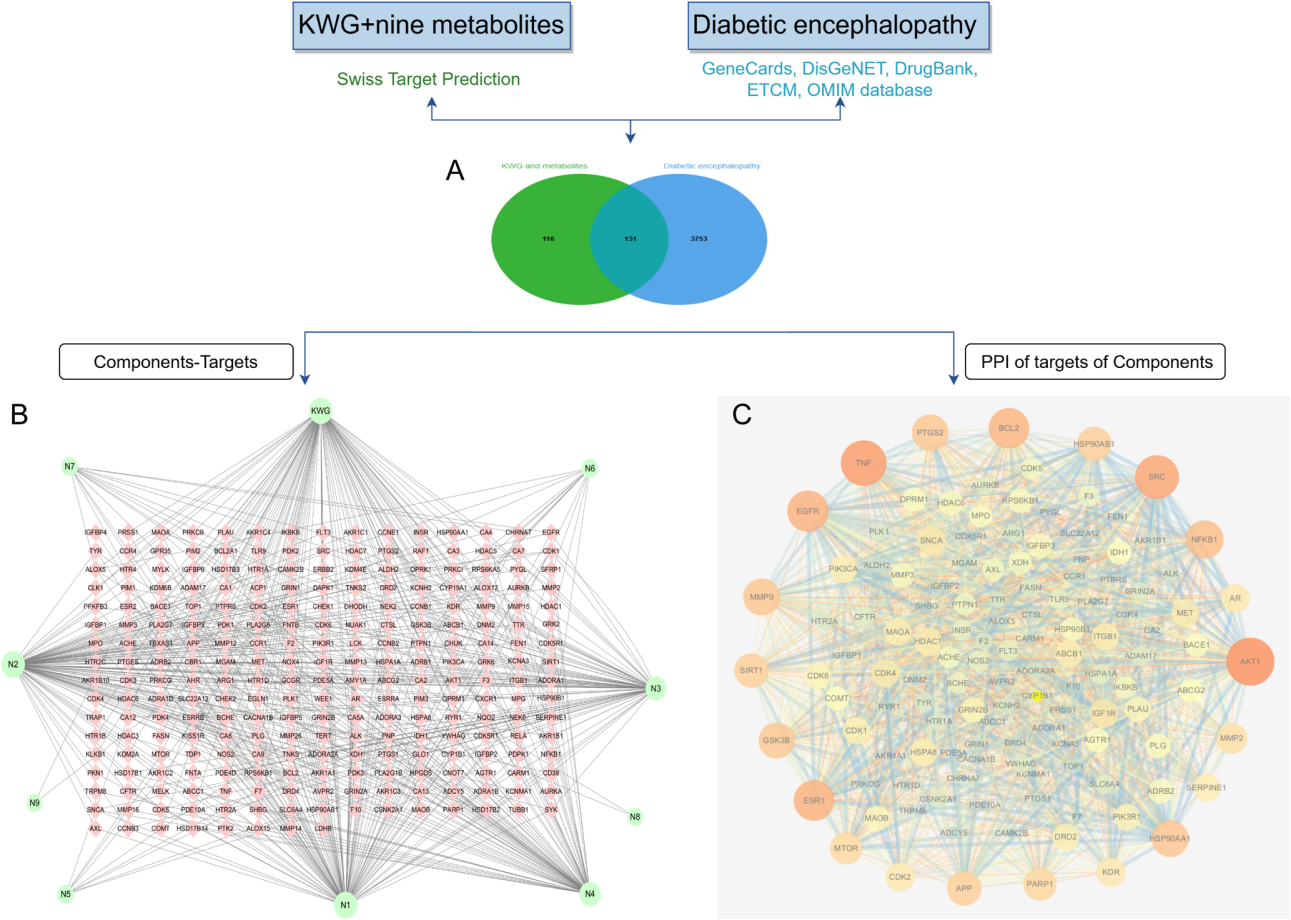
Schematic diagram of network pharmacology (A: Venn diagrams of 131 common targets; B: The ingredient‐target network of KWG and its active metabolites, and the component size was correlated to the number of targets involved; C: PPI network of targets, and the size of the target circle was related to the target degree value).

Nine metabolites (N1–N9) with high gastrointestinal absorption and drug‐likeness were selected through Swiss ADME, which are shown in Table 2. The targets corresponding to KWG and its 9 metabolites were searched, duplicate items were deleted, and a total of 248 targets were obtained. Through searching the databases of GeneCards, DisGeNET, DrugBank, ETCM, and OMIM, 3884 predicted targets for DE were obtained. Using Venny 2.1.0 to analyze the DE disease targets of the active ingredients, 131 intersection targets were obtained. The intersection targets were submitted to the String11.0 database to obtain the protein interaction network diagram of the intersection targets. By analyzing the degree values of three topological parameters in the network, the main targets were screened out from this PPI network; the top five core targets (AKT1, TNF, SRC, EGFR, ESR1) related to DE and core components were selected for macromolecular docking. The results are shown in Figure 6 (by Figdraw) and Table 3.

**TABLE 2:** Target information of nine metabolites of KWG.

No.	Target number	Structure
KWG	101	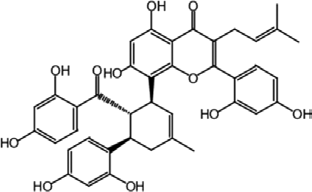
N1 (M25)	105	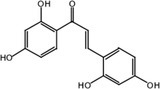
N2 (M34)	102	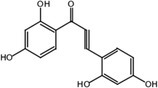
N3 (M35)	73	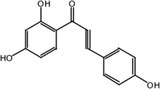
N4 (M26)	74	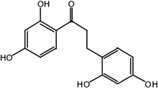
N5 (M27)	9	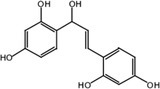
N6 (M49)	13	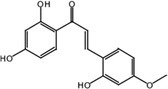
N7 (M50)	14	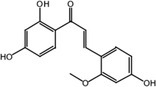
N8 (M51)	2	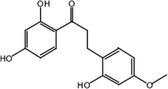
N9 (M52)	101	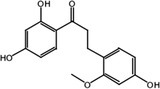

**TABLE 3:** The top five diabetic encephalopathy‐related disease targets.

No.	Targets	Betweenness	Closeness	Degree
1	AKT1	1808.811	0.741177	82
2	TNF	1495.907	0.715909	76
3	SRC	1250.302	0.692308	71
4	EGFR	666.3804	0.670213	65
5	ESR1	768.7154	0.666667	64

**FIGURE 7:**
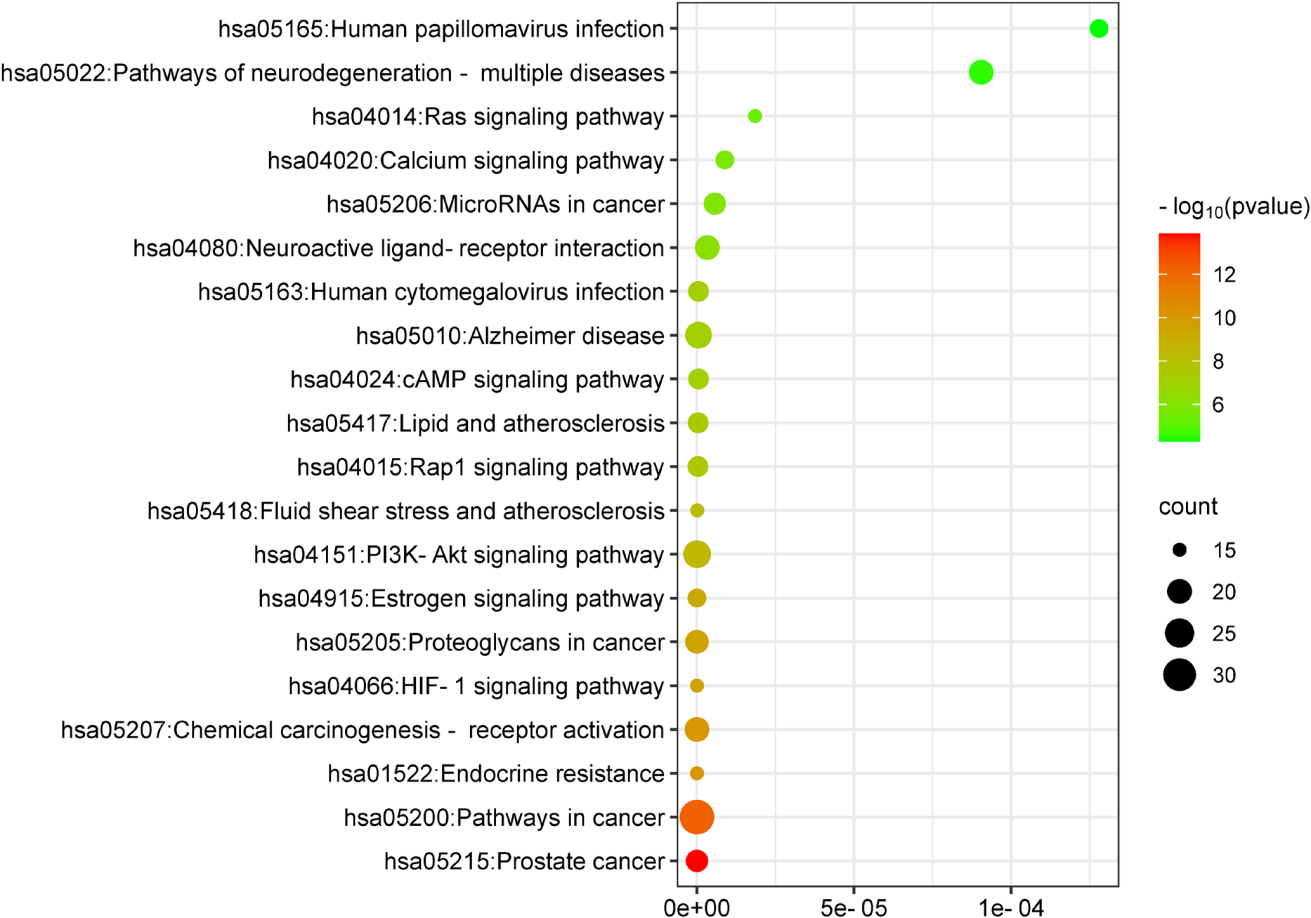
Top 20 KEGG pathway analysis of key targets. (The bubble color ranged from orange to green, representing the decreasing −log10 value (*p* value) of the pathway, and the size of the circle of the bubble indicate the number of target genes associated with each pathway.)

**FIGURE 8:**
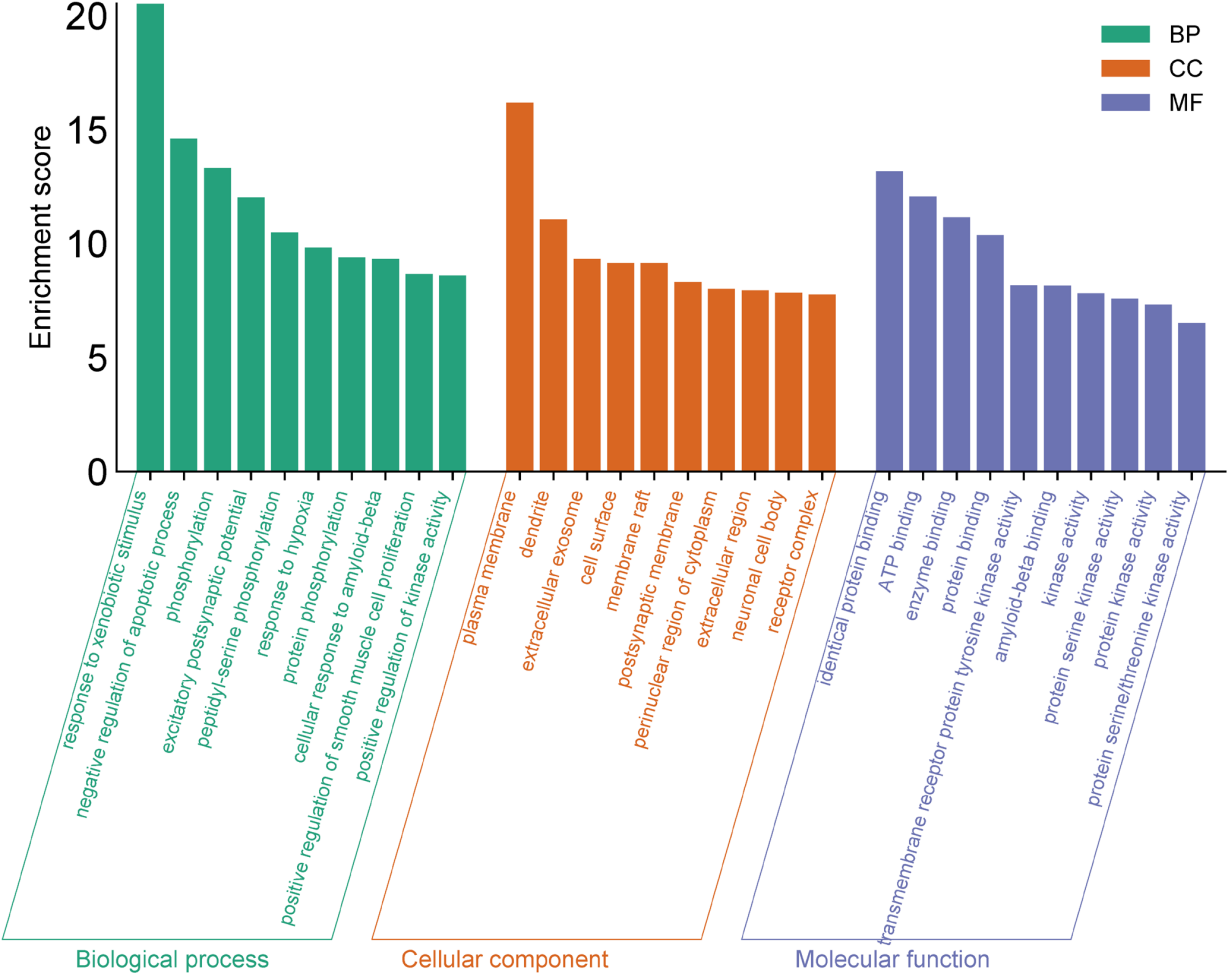
Top 10 GO terms in BP, CC, and MF categories.

To gain a comprehensive understanding of the mechanisms by which KWG and nine metabolites exert their effects on DE at the systemic level, we conducted KEGG and GO enrichment analyses on 131 intersecting targets. The KEGG enrichment analysis revealed that these targets were significantly associated with 120 signaling pathways (*p* < 0.05), including the PI3K–Akt signaling pathway, Alzheimer's disease, pathways of neurodegeneration across multiple diseases, and neuroactive ligand–receptor interactions, among others (Figure 7). Furthermore, the GO enrichment results indicated that the targets were enriched in 455 biological processes (BPs), 99 cellular components (CCs), and 147 molecular functions (MFs) (*p* < 0.05). As depicted in Figure 8, within the BP category, the primary enrichments included responses to xenobiotic stimuli, negative regulation of the apoptotic process, phosphorylation, and excitatory postsynaptic potential. In the CC category, the main enrichments were observed in the plasma membrane, dendrites, extracellular exosomes, and cell surfaces. In the MF category, significant enrichments included identical protein binding, ATP binding, amyloid‐beta binding, and protein binding. The findings from the KEGG and GO enrichment analyses suggest that these targets may be extensively involved in BPs related to neurodegenerative diseases, cell signal transduction, regulation of cell survival, and neural signal transmission. Notably, the enrichment of the PI3K–Akt signaling pathway and Alzheimer's disease‐related pathways indicates that these targets may play a crucial role in the context of neurodegenerative diseases.

**FIGURE 9:**
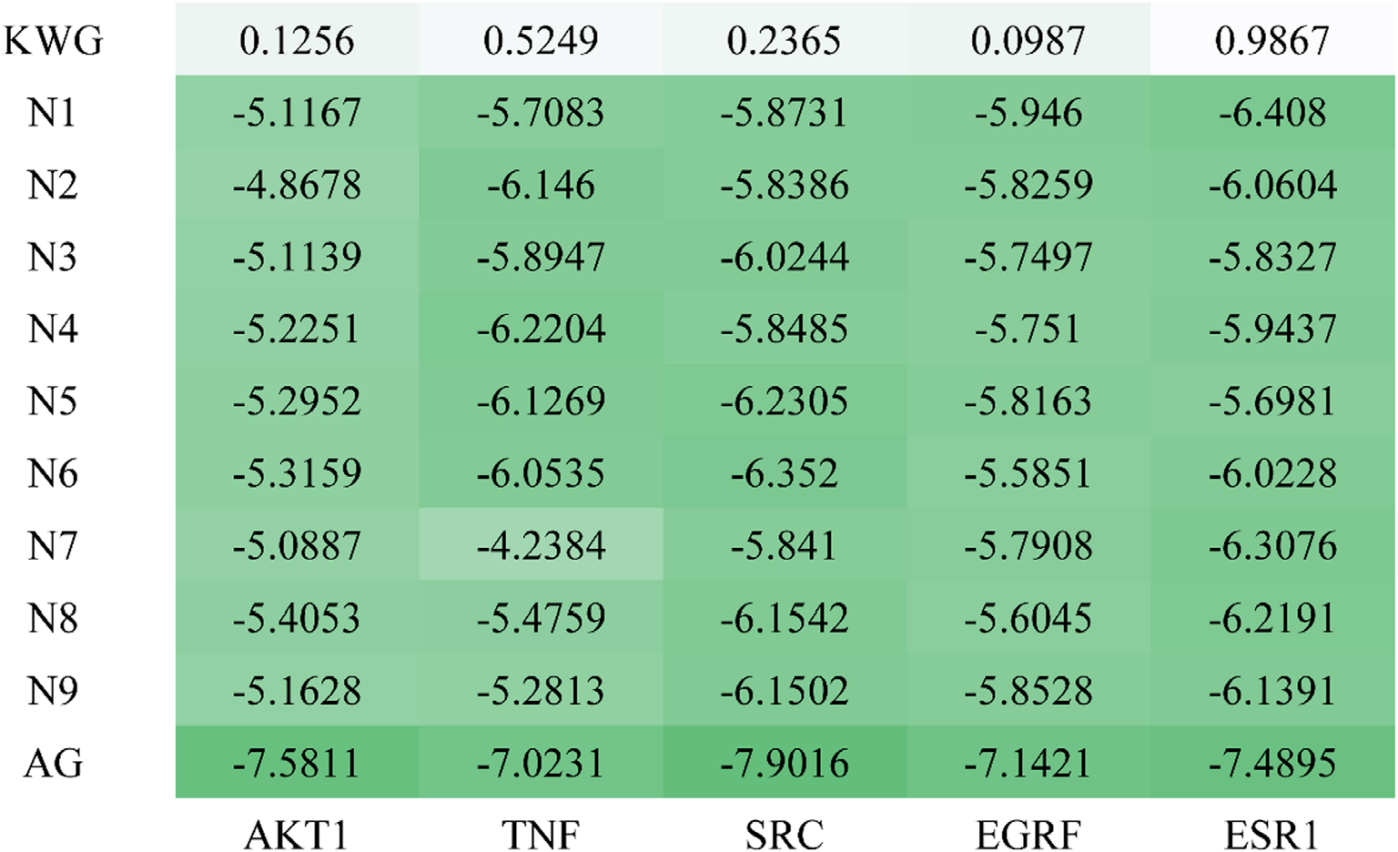
Heat maps of molecular docking results. (The greener the color, the smaller the corresponding value, indicating the greater the docking strength.)

**FIGURE 10:**
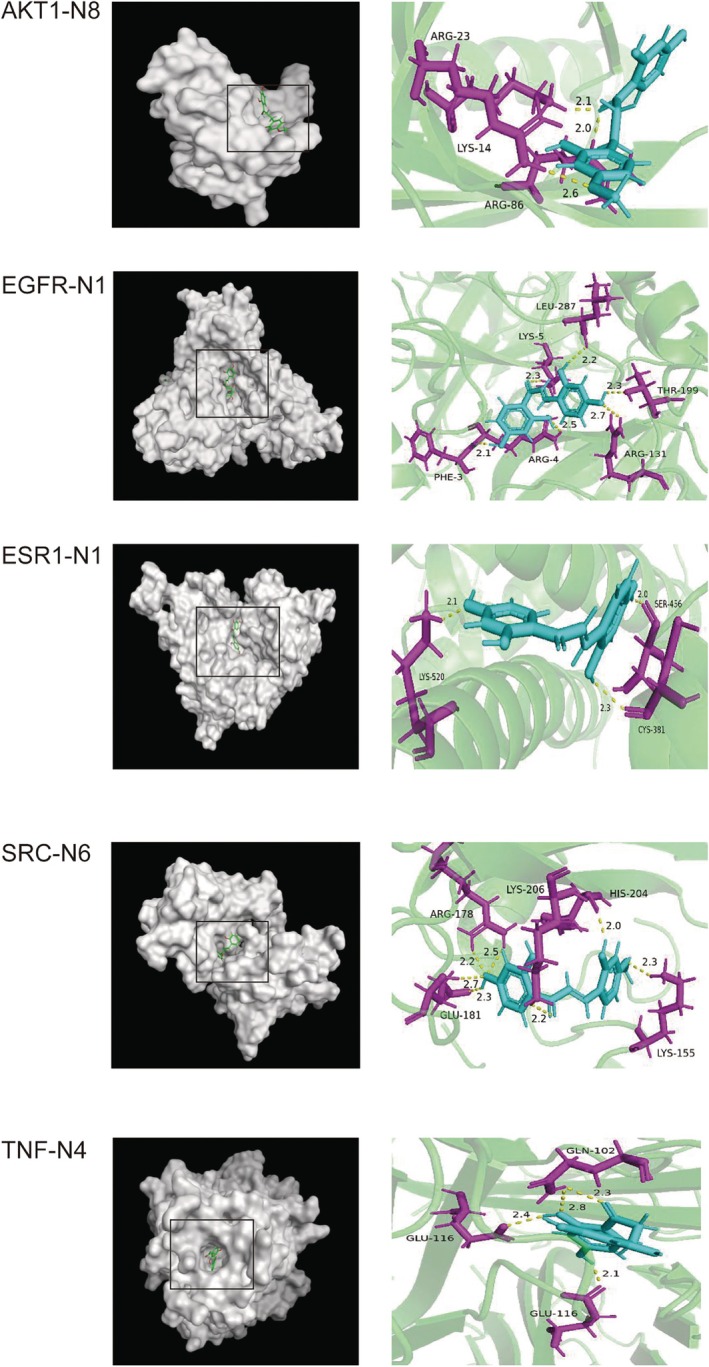
The 3D interaction diagrams of AKT1‐N8, EGRF‐N1, ESR1‐N1, SRC‐N6, TNF‐N4.

In molecular docking studies, it is generally accepted that a binding energy of less than −4.25 kcal/mol between the ligand and protein indicates specific binding activity, while a binding energy of less than −5.0 kcal/mol suggests good binding activity (Yang et al. 2023). The binding energies of AGEs inhibitor aminoguanidine with AKT1, TNF, SRC, EGFR, ESR1, and AKT1 were −7.5811, −7.0231, −7.9016, −7.1421, and −7.4895 kcal/mol, shown in Figure 9 and Figure S1. This indicated that the AGEs inhibitor aminoguanidine exhibited strong binding affinity towards these targets, which were considered potential therapeutic candidates for DE. As shown in Figures 9 and 10, the binding energy of KWG to target proteins such as AKT1, TNF, SRC, EGFR, and ESR1 was all greater than 0, indicating that KWG is unlikely to bind to these core targets. In contrast, the binding energies of its metabolites to these same targets were all below −4.8678 kcal/mol. These findings suggest that the active form of KWG in biological systems is not the original compound but rather its active metabolites. This indicates that a substance with low oral bioavailability can be transformed into several metabolites with improved oral bioavailability to exert its effects.

### 3.6 Results of Dynamics Simulations

**FIGURE 11:**
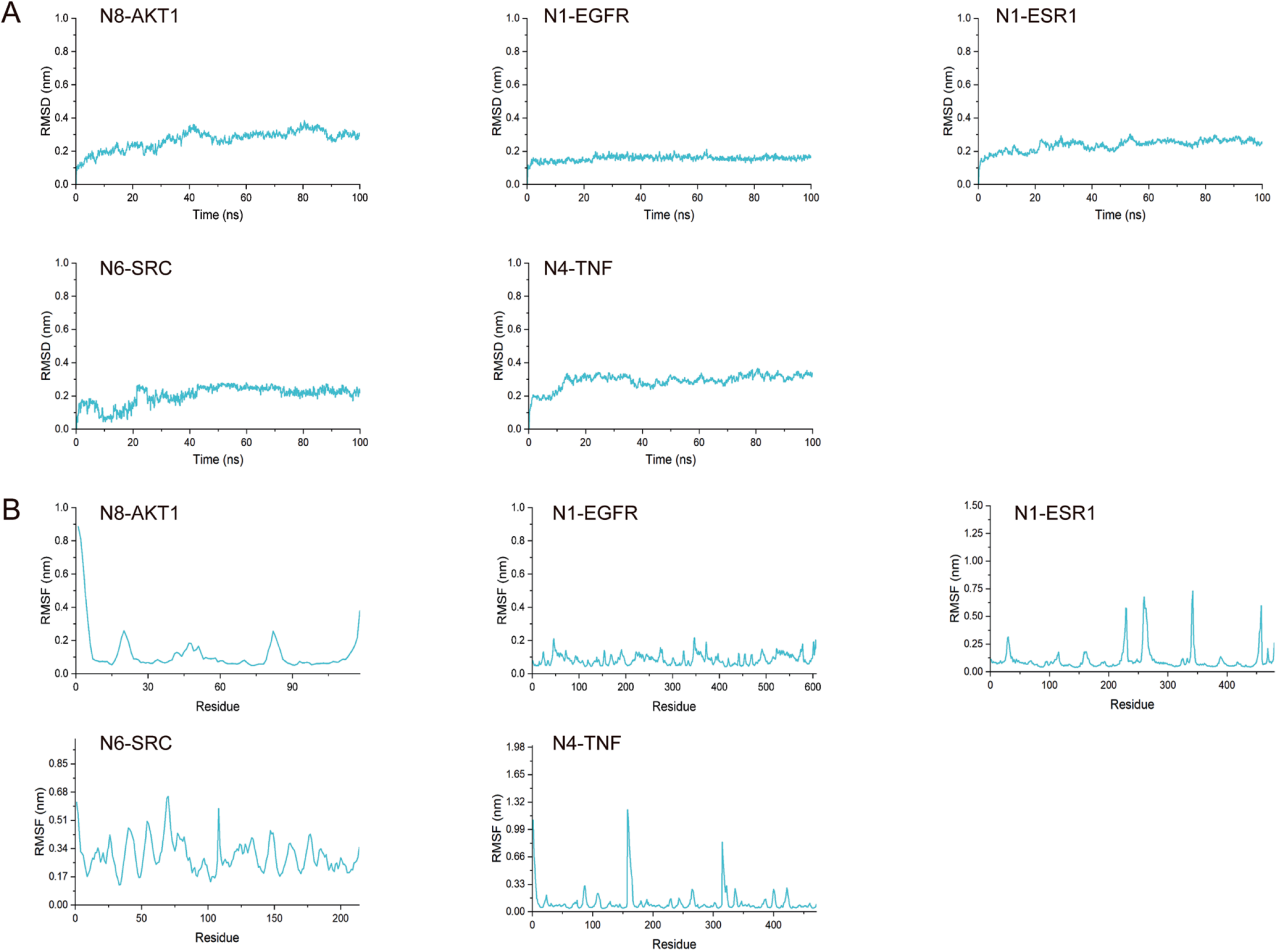
The RMSD (A) and RMSF (B) of five complexes.

**FIGURE 12:**
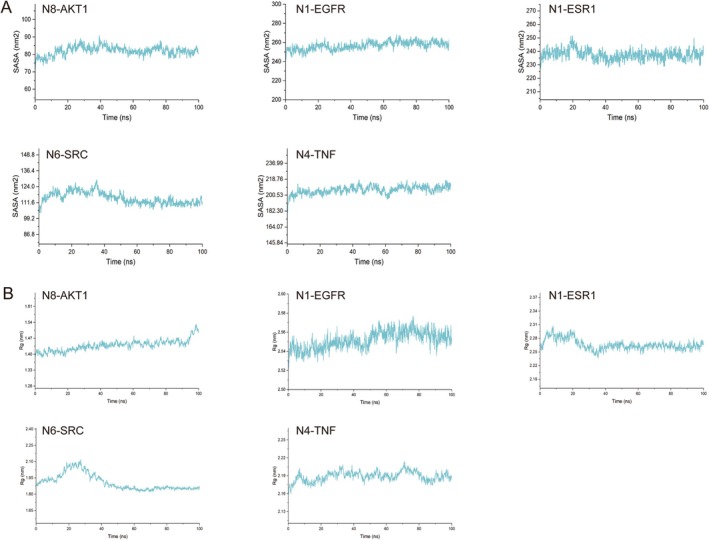
The SASA (A) and Rg (B) of five complexes.

**FIGURE 13:**
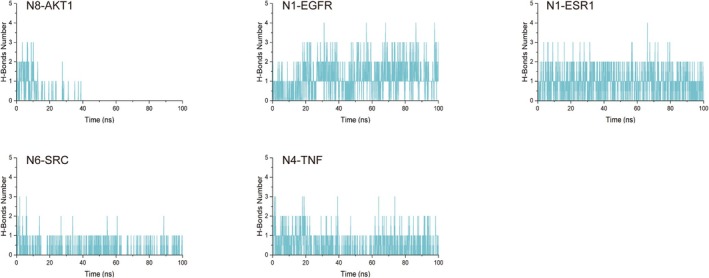
The Hbond of five complexes.

In the results of molecular docking, five best complexes—protein complexes (N8‐AKT1, N4‐TNF, N6‐SRC, N1‐EGFR, and N1‐ESR1)—were subjected to a 100‐ns MDS analysis. The dynamic trajectories of the five complexes were analyzed using RMSD/RMSF/SASA/Rg. From Figure 11A, it could be seen that the RMSD deviation of the complexes was basically within the 0.2‐nm range, and the curves were relatively stable, indicating that the five complexes had a stability level. We determined the residue fluctuations of the five complexes through RMSF analysis and understood the residues that had significant changes during the MDS process (Figure 11B). We understood the hydrophobicity and surface state of the protein through SASA analysis. During the MDS process, the SASA value of the five complexes was relatively stable (Figure 12A). The Rg curve showed the compactness of the entire system, as shown in Figure 12B; the stable rotational radius of the five complexes indicated the stability of the protein structure. Hydrogen bonds were an important part of the interaction between the protein and the ligand. By tracking the change in the number of hydrogen bonds over time, we gained a deeper understanding of the dynamic process of the ligand binding to the protein. All five complexes have two or more hydrogen bonds, which made the complexes more stable (Figure 13).

**FIGURE 14:**
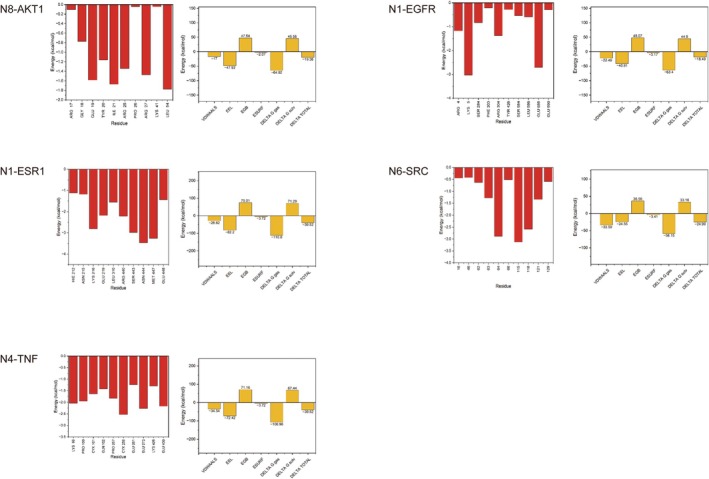
Amino acid residues involved in binding and binding energies of five complexes.

The binding free energy between the compound and the target protein was calculated using the MM/GBSA method. According to the calculation results (Figure 14), the active metabolite had affinity for the protein; the strongest binding forces of N8‐AKT1, N1‐EGFR, N1‐ESR1, N6‐SRC, and N4‐TNF were at −19.36, −18.49, −39.52, −24.99 and −39.52 kcal/mol, respectively. During the molecular dynamics simulation of N8‐AKT1, LEU 54 and ILE 21 formed hydrophobic pockets that stabilized N8 binding. ARG 27, ARG 25, ARG 17, LYS 41, and GLU 19 might interact electrostatically with N8, while TYR 20 and GLY 18 contributed to hydrogen bond formation. These residues synergistically enhanced N8‐AKT1 binding. For N1‐EGFR, ARG 304 and ARG 4 formed electrostatic interactions with N1, reducing binding free energy. LEU 586 and PHE 303 stabilized N1 via hydrophobic effects, and SER 284, SER 584, and TYR 426 provided additional stability through hydrogen bonds. In N1‐ESR1, ARG 440 and LYS 216 reduced binding free energy via electrostatic interactions, while ASN 444 and ASN 215 stabilized the complex through hydrogen bonds. MET 447 and LEU 310 lowered solvation energy by reducing solvent contact. For N6‐SRC, electrostatic interactions between ARG/LYS and N6 reduced binding free energy, and aromatic residues like PHE and TYR enhanced binding through π–π stacking or cation–π interactions. Hydrophilic GLU residues contributed to hydrogen bond networks, and hydrophobic ILE/LEU residues reduced solvation free energy. During N4‐TNF simulation, GLU and LYS residues formed salt bridges, reducing binding free energy, and CYX residues formed disulfide bonds to stabilize the protein structure.

## 4 Discussion

This study examined the metabolism of KWG both in vivo (in plasma, bile, urine, and feces) and in vitro (using rat liver microsomes and intestinal flora). A total of 56 metabolites were identified in vivo, while 52 metabolites were detected in vitro. Notably, 37 metabolites were generated by rat intestinal flora compared to 24 in liver microsomes, underscoring the superior metabolic capacity of gut microbiota in transforming KWG into bioactive derivatives (Du et al. 2014). The distribution pattern of metabolites—14 in plasma, 7 in bile, and 3 in urine, with complete detection in feces—highlighted predominant intestinal metabolism and absorption after oral administration. Importantly, while KWG itself was undetectable in systemic circulation, its gut microbiota‐derived metabolites were present in the bloodstream, suggesting that intestinal biotransformation was critical for its pharmacological activity. Emerging evidence further posits that flavonoids like KWG may reciprocally modulate gut microbiota, exhibiting prebiotic potential. For instance, Yuan et al. demonstrated that mulberry polyphenols selectively enrich beneficial taxa such as Lactobacillus and Bifidobacterium while suppressing pathogens, a hallmark of prebiotic action (Yuan and Zhao 2017). Similarly, Guo et al. observed that KWG preserves gut epithelial integrity under inflammatory conditions, indirectly fostering a symbiotic microbial niche (Guo et al. 2016). These findings suggest a dual role for KWG: its metabolites mediate neuroprotection, while the parent compound may stabilize gut ecology to amplify therapeutic outcomes.

It is widely acknowledged that the orally administrated Traditional Chinese Medicine (TCM) herbal components frequently are not absorbed into the blood directly, but rather enter the intestine and are subsequently decomposed by intestinal flora (Lin et al. 2021). In certain herbal medicines, the bio‐conversion mediated by gut microbiota plays a crucial role in the manifestation of their therapeutic effects or toxicities in vivo. For example, the active form of platycodin D that exerts the effect of relieving cough and eliminating phlegm is its intestinal flora metabolite (Zhong et al. 2023). In this study, we found that KWG was primarily metabolized by gut microbiota in vivo; however, the potential activity of these metabolites remained to be elucidated. We subsequently used Swiss ADME to conduct an activity screening of all in vivo metabolites, identifying a total of nine active metabolites, all of which were detected in plasma, indicating that they could be absorbed into the bloodstream. Preliminary research suggested that due to the larger molecular weight of the parent compound KWG, its bioavailability to neurons was relatively low, limiting its permeability across biological barriers, including the blood–brain barrier (BBB) (Gan et al. 2021). In contrast, the metabolites that were absorbed into the bloodstream possess smaller molecular weights, which might facilitate their ability to cross biological barriers, including the BBB, thereby exerting therapeutic effects on DE. Preliminary calculations and predictions conducted using SwissADME suggested that compounds N1, N4, N6, and N8 might possess BBB permeability and were likely to serve as substrates for P‐glycoprotein (P‐gp). These results were included in Table S3.

Active metabolites interact with their targets to produce final effects, which is why molecular docking is employed to investigate the binding affinity between these metabolites and their respective targets. Typically, a binding energy below 0 suggests that the ligand can bind spontaneously to the acceptor molecule; lower binding energies generally indicate stronger affinities and enhanced activities (Long et al. 2022). The binding energy of KWG to the core target was around 0 kcal/mol, and the binding energy of the metabolites to the core target was all below −4.8678 kcal/mol. Moreover, the targets of these active metabolites were different from those of KWG. The research results further indicated that the prototype drug might produce a large number of metabolites after administration, and the specific molecular form in which the therapeutic effect was exerted may be by the metabolites produced by the prototype. These results not only clarify the phenomenon of low oral bioavailability yet high activity of TCM, but also illuminate the phenomenon that one component of TCM acts on multiple targets.

**FIGURE 15:**
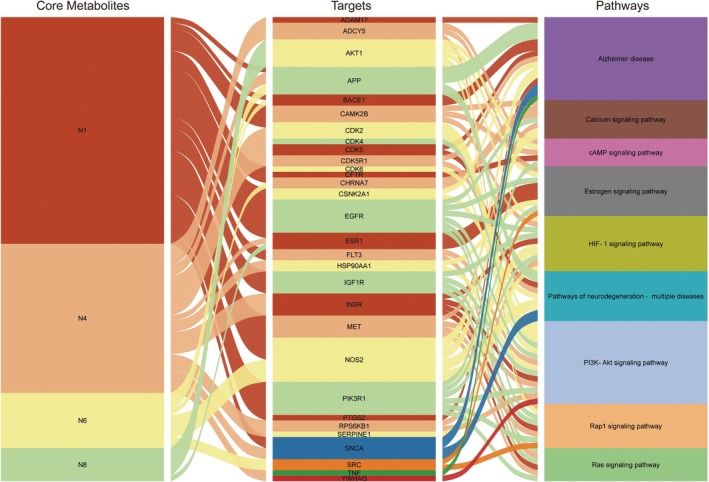
Regulatory network of core metabolites, target molecules, and associated signaling pathways (edges: arrows indicate regulatory interactions).

Through integrative network pharmacology and molecular docking analyses, we identified four core bioactive metabolites of N1, N4, N6, and N8 that exhibit potent binding affinities to key therapeutic targets—AKT1, TNF, SRC, EGFR, and ESR1central to DE pathogenesis. These targets collectively modulate interconnected pathways critical for neuroprotection: (1) AKT1, a pivotal regulator of the PI3K/Akt signaling pathway, sustains neuronal survival and synaptic plasticity, with its dysfunction implicated in diabetes‐associated cognitive decline (Wang and Zhao 2016); (2) TNF drives neuroinflammation and oxidative stress, exacerbating BBB disruption in DE models (Gireesh et al. 2009; Zhu et al. 2018); (3) SRC kinase activation promotes microglial‐mediated cytokine release and neurodegeneration under hyperglycemic conditions (Yang et al. 2020); (4) EGFR contributes to neuronal apoptosis and glial hyperactivation (Kimura et al. 2013); and (5) ESR1 (estrogen receptor α) mediates anti‐inflammatory and antioxidant responses, counteracting diabetic cognitive impairment (Bae et al. 2000). Network topology and KEGG enrichment revealed these targets as hubs bridging oxidative stress, synaptic dysfunction, and hormonal regulation in DE. Notably, the four metabolites orchestrate multi‐target regulation of the PI3K–Akt signaling pathway, Alzheimer's disease‐related cascades, and neurodegeneration networks (Figure 15), aligning with prior evidence of KWG's neuroprotection via PI3K/Akt/GSK3αβ modulation (Gan et al. 2021) and broader recognition of natural products targeting this axis (Wang and Zhao 2016; Cui et al. 2022). These findings illuminate KWG's metabolic activation as a prodrug, with metabolites engaging a synergistic, multi‐pathway mechanism—advancing TCM pharmacology by linking biotransformation to targeted neuroprotection in diabetic complications.

The interaction among binding energy, drug similarity, and bioavailability is crucial for evaluating the therapeutic potential of KWG metabolites (Gayathiri et al. 2022). The metabolites N1, N4, N6, and N8 exhibited robust drug‐likeness and bioavailability potential, supported by their adherence to Lipinski's Rule of Five (Prakash et al. 2022) with molecular weights (271–287 Da) and Clog P values (2.05672–3.10321) within optimal ranges for oral absorption and BBB permeability. SwissADME predictions further confirmed their BBB‐penetrant potential, aligning with physicochemical profiles typical of CNS‐targeted agents (Gayathiri, Prakash, Pratheep, et al. 2024). MM/GBSA‐derived binding free energies revealed strong affinities for DE‐related targets: N1‐ESR1 (−39.52 kcal/mol) and N4‐TNF (−39.52 kcal/mol) demonstrated the highest stability, driven by synergistic electrostatic interactions (ARG440/LYS216 in N1‐ESR1; GLU/LYS salt bridges in N4‐TNF) and hydrogen bonding networks (ASN444/ASN215 in N1‐ESR1). N6‐SRC (−24.99 kcal/mol) leveraged π–π stacking (PHE/TYR) and hydrophilic interactions (GLU), while N8‐AKT1 (−19.36 kcal/mol) relied on hydrophobic pockets (LEU54/ILE21) and transient hydrogen bonds (TYR20/GLY18). Molecular dynamics simulations highlighted reduced solvation energy in N1‐ESR1 (MET447/LEU310) and N8‐AKT1 (ARG27/LYS41), critical for metabolic stability and passive BBB diffusion (Gurunathan, Thangaraj, Das, and Kim 2023). These multi‐mechanistic interactions—balancing hydrophobicity, polarity, and electrostatic forces—mirror strategies employed in high‐bioavailability CNS drugs. Although the calculated data are consistent with previous studies on flavonoid‐derived neuroprotective agents, in order to promote their clinical application, human bioavailability studies are crucial for verifying the absorption, metabolism, and retention of these key metabolites in the systemic circulation and target tissues (such as the brain). Furthermore, in the development of functional foods, preparations rich in KWG, such as microcapsule supplements, can effectively alleviate the progression of DE through their metabolites. However, this requires rigorous safety and stability evaluations under simulated gastrointestinal conditions to ensure clinical applicability (Gurunathan, Thangaraj, and Kim 2023). Future work will focus on in vitro BBB models and in vivo bioavailability studies to link these predictive insights with experimental evidence (Gayathiri, Prakash, Ahamed, et al. 2024) and provide a basis for the clinical application and transformation of KWG.

## 5 Conclusions

This study explored the metabolism of Kuwanon G (KWG) in rats, identifying 56 in vivo and 52 in vitro metabolites, with gut microbiota playing a key role in biotransformation. Using network pharmacology and molecular docking, we found that KWG's metabolites, particularly N1, N4, N6, and N8, exhibit strong binding to DE‐related targets (AKT1, TNF, SRC, EGFR, ESR1), suggesting their therapeutic potential.

These metabolites modulate pathways such as PI3K–Akt, indicating their role in neuroprotection. The findings highlight KWG's potential as a functional food or nutraceutical for managing DE, with future research needed to validate these results and explore gut microbiota's role in enhancing bioavailability.

## Author Contributions

**Yuqian Zhang:** conceptualization (equal), project administration (equal), writing – original draft (equal). **Siying Zhang:** data curation (equal). **Haiying Niu:** formal analysis (equal). **Weiwei Xie:** methodology (equal). **Yuxin Tan:** formal analysis (equal). **Deqiang Li:** investigation (equal). **Yiran Jin:** writing – review and editing (equal).

## Conflicts of Interest

The authors declare no conflicts of interest.

## Supporting information


**Figure S1.** The 3D interaction diagrams of AGE inhibitor aminoguanidine with AKT1, EGRF, ESR1, SRC, and TNF.

**Table S1.** The results of Gene Ontology.

**Table S2.** The results of Kyoto Encyclopedia of Genes and Genomes.

**Table S3.** Assessment results of BBB permeability and P‐gp substrate properties of active metabolites via SwissADME.

## Data Availability

The data that support the findings of this study are available on request from the corresponding author.
