# Supplementary material for: Metabolite‐Based Network Pharmacology, Molecular Docking, and Dynamics Simulations to Preliminarily Verify Treating Diabetic Encephalopathy Effect of Kuwanon G
Source: Food Sci Nutr. 2025 Jun 7;13(6):e70392. doi: 10.1002/fsn3.70392 (PMC12144589; doi:10.1002/fsn3.70392)
Supplement: Supplementary file 1 — Figure S1. The 3D interaction diagrams of AGE inhibitor aminoguanidine with AKT1, EGRF, ESR1, SRC, and TNF. [file FSN3-13-e70392-s004.docx]

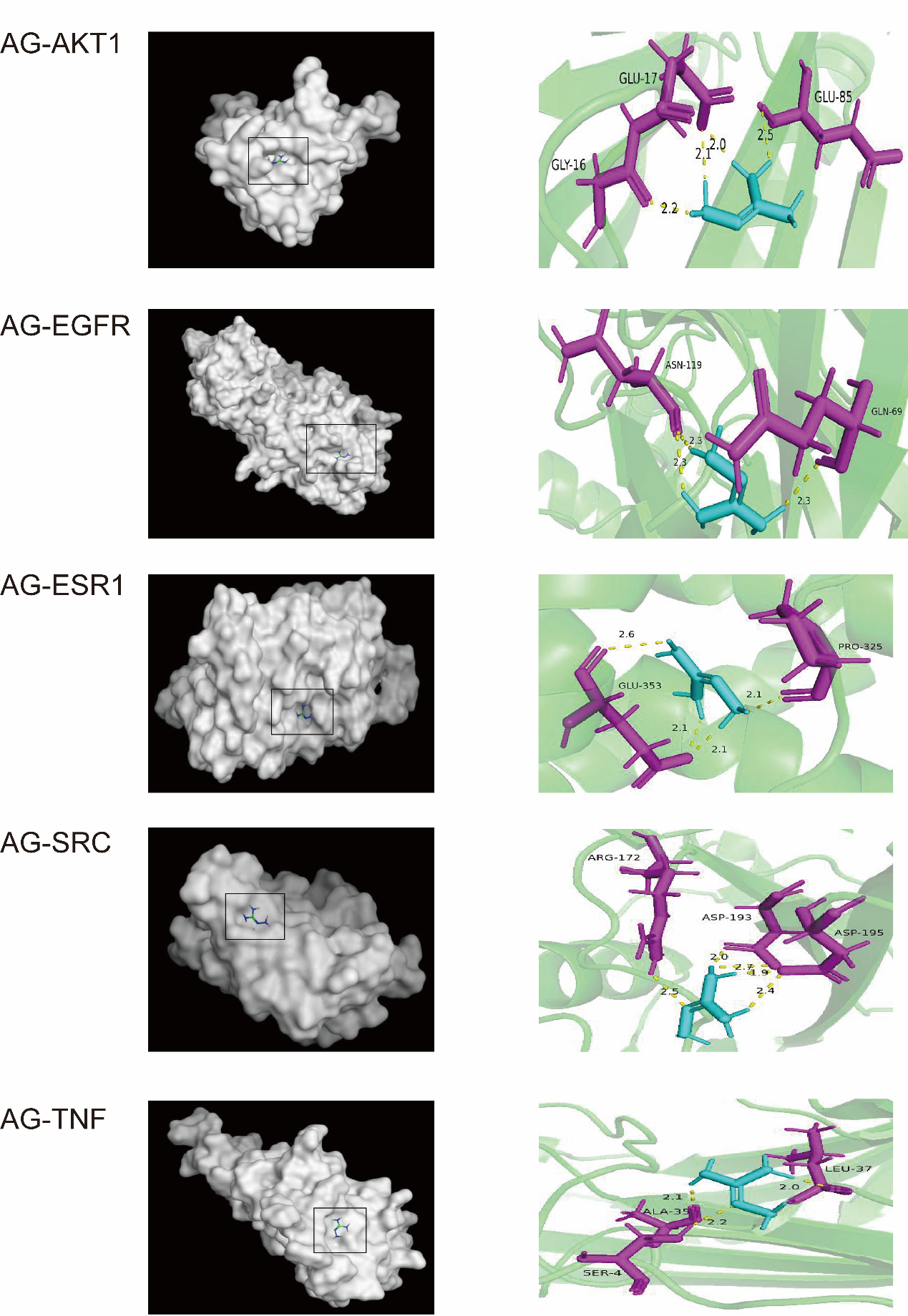


Figure S1. The 3D interaction diagrams of AGEs inhibitor aminoguanidine with AKT1, EGRF, ESR1, SRC and TNF.
