# Supplementary material for: Metabolite‐Based Network Pharmacology, Molecular Docking, and Dynamics Simulations to Preliminarily Verify Treating Diabetic Encephalopathy Effect of Kuwanon G
Source: Food Sci Nutr. 2025 Jun 7;13(6):e70392. doi: 10.1002/fsn3.70392 (PMC12144589; doi:10.1002/fsn3.70392)
Supplement: Supplementary file 2 — Table S1. The results of Gene Ontology. [file FSN3-13-e70392-s002.docx]

Table S1 The results of Gene Ontology

| Category | Term | Count | PValue | FDR |
| --- | --- | --- | --- | --- |
| GOTERM_MF_DIRECT | GO:0042802~identical protein binding | 43 | 6.89E-14 | 3.53E-11 |
| GOTERM_MF_DIRECT | GO:0005524~ATP binding | 39 | 8.68E-13 | 2.22E-10 |
| GOTERM_MF_DIRECT | GO:0019899~enzyme binding | 20 | 7.04E-12 | 1.20E-09 |
| GOTERM_MF_DIRECT | GO:0005515~protein binding | 119 | 4.24E-11 | 5.43E-09 |
| GOTERM_MF_DIRECT | GO:0004714~transmembrane receptor protein tyrosine kinase activity | 8 | 6.86E-09 | 6.05E-07 |
| GOTERM_MF_DIRECT | GO:0001540~amyloid-beta binding | 10 | 7.09E-09 | 6.05E-07 |
| GOTERM_MF_DIRECT | GO:0016301~kinase activity | 13 | 1.51E-08 | 1.11E-06 |
| GOTERM_MF_DIRECT | GO:0106310~protein serine kinase activity | 16 | 2.59E-08 | 1.66E-06 |
| GOTERM_MF_DIRECT | GO:0004672~protein kinase activity | 14 | 4.84E-08 | 2.75E-06 |
| GOTERM_MF_DIRECT | GO:0004674~protein serine/threonine kinase activity | 15 | 3.05E-07 | 1.56E-05 |
| GOTERM_CC_DIRECT | GO:0005886~plasma membrane | 81 | 6.53E-17 | 1.97E-14 |
| GOTERM_CC_DIRECT | GO:0030425~dendrite | 21 | 8.60E-12 | 1.30E-09 |
| GOTERM_CC_DIRECT | GO:0070062~extracellular exosome | 41 | 4.68E-10 | 4.41E-08 |
| GOTERM_CC_DIRECT | GO:0009986~cell surface | 22 | 7.28E-10 | 4.41E-08 |
| GOTERM_CC_DIRECT | GO:0045121~membrane raft | 14 | 7.30E-10 | 4.41E-08 |
| GOTERM_CC_DIRECT | GO:0045211~postsynaptic membrane | 13 | 4.85E-09 | 2.44E-07 |
| GOTERM_CC_DIRECT | GO:0048471~perinuclear region of cytoplasm | 22 | 9.79E-09 | 4.22E-07 |
| GOTERM_CC_DIRECT | GO:0005576~extracellular region | 37 | 1.12E-08 | 4.24E-07 |
| GOTERM_CC_DIRECT | GO:0043025~neuronal cell body | 16 | 1.42E-08 | 4.77E-07 |
| GOTERM_CC_DIRECT | GO:0043235~receptor complex | 13 | 1.72E-08 | 5.18E-07 |
| GOTERM_BP_DIRECT | GO:0009410~response to xenobiotic stimulus | 25 | 3.04E-21 | 6.43E-18 |
| GOTERM_BP_DIRECT | GO:0043066~negative regulation of apoptotic process | 26 | 2.52E-15 | 2.67E-12 |
| GOTERM_BP_DIRECT | GO:0016310~phosphorylation | 27 | 4.90E-14 | 3.45E-11 |
| GOTERM_BP_DIRECT | GO:0060079~excitatory postsynaptic potential | 12 | 9.42E-13 | 4.98E-10 |
| GOTERM_BP_DIRECT | GO:0018105~peptidyl-serine phosphorylation | 14 | 3.29E-11 | 1.39E-08 |
| GOTERM_BP_DIRECT | GO:0001666~response to hypoxia | 14 | 1.49E-10 | 5.24E-08 |
| GOTERM_BP_DIRECT | GO:0006468~protein phosphorylation | 18 | 4.07E-10 | 1.23E-07 |
| GOTERM_BP_DIRECT | GO:1904646~cellular response to amyloid-beta | 9 | 4.71E-10 | 1.24E-07 |
| GOTERM_BP_DIRECT | GO:0048661~positive regulation of smooth muscle cell proliferation | 9 | 2.17E-09 | 5.08E-07 |
| GOTERM_BP_DIRECT | GO:0033674~positive regulation of kinase activity | 9 | 2.52E-09 | 5.33E-07 |
