# Supplementary material for: Metabolite‐Based Network Pharmacology, Molecular Docking, and Dynamics Simulations to Preliminarily Verify Treating Diabetic Encephalopathy Effect of Kuwanon G
Source: Food Sci Nutr. 2025 Jun 7;13(6):e70392. doi: 10.1002/fsn3.70392 (PMC12144589; doi:10.1002/fsn3.70392)
Supplement: Supplementary file 3 — Table S2. The results of Kyoto Encyclopedia of Genes and Genomes. [file FSN3-13-e70392-s003.docx]

Table S2 The results of Kyoto Encyclopedia of Genes and Genomes

| term | FDR | PValue | Count |
| --- | --- | --- | --- |
| hsa05200:Pathways in cancer | 4.03E-11 | 5.76E-13 | 33 |
| hsa04151:PI3K-Akt signaling pathway | 4.83E-08 | 3.45E-09 | 23 |
| hsa05010:Alzheimer disease | 5.11E-07 | 7.10E-08 | 22 |
| hsa05207:Chemical carcinogenesis - receptor activation | 2.46E-09 | 8.78E-11 | 20 |
| hsa04080:Neuroactive ligand-receptor interaction | 3.31E-06 | 5.92E-07 | 20 |
| hsa05022:Pathways of neurodegeneration - multiple diseases | 9.05E-05 | 3.10E-05 | 20 |
| hsa05205:Proteoglycans in cancer | 5.92E-09 | 2.96E-10 | 19 |
| hsa05215:Prostate cancer | 1.93E-12 | 1.38E-14 | 18 |
| hsa05206:MicroRNAs in cancer | 5.69E-06 | 1.18E-06 | 18 |
| hsa04015:Rap1 signaling pathway | 3.20E-07 | 2.97E-08 | 17 |
| hsa05417:Lipid and atherosclerosis | 3.87E-07 | 3.87E-08 | 17 |
| hsa04024:cAMP signaling pathway | 5.11E-07 | 7.30E-08 | 17 |
| hsa05163:Human cytomegalovirus infection | 5.11E-07 | 7.30E-08 | 17 |
| hsa04915:Estrogen signaling pathway | 9.57E-09 | 6.10E-10 | 16 |
| hsa04020:Calcium signaling pathway | 8.86E-06 | 1.96E-06 | 16 |
| hsa05165:Human papillomavirus infection | 1.28E-04 | 5.05E-05 | 16 |
| hsa01522:Endocrine resistance | 2.12E-09 | 6.05E-11 | 15 |
| hsa04066:HIF-1 signaling pathway | 5.30E-09 | 2.27E-10 | 15 |
| hsa05418:Fluid shear stress and atherosclerosis | 9.01E-08 | 7.08E-09 | 15 |
| hsa04014:Ras signaling pathway | 1.85E-05 | 4.63E-06 | 15 |
