# Supplementary material for: Metabolite‐Based Network Pharmacology, Molecular Docking, and Dynamics Simulations to Preliminarily Verify Treating Diabetic Encephalopathy Effect of Kuwanon G
Source: Food Sci Nutr. 2025 Jun 7;13(6):e70392. doi: 10.1002/fsn3.70392 (PMC12144589; doi:10.1002/fsn3.70392)
Supplement: Supplementary file 4 — Table S3. Assessment results of BBB permeability and P‐gp substrate properties of active metabolites via SwissADME. [file FSN3-13-e70392-s001.docx]

Table S3 Assessment Results of BBB Permeability and P-gp Substrate Properties of Active Metabolites via SwissADME

| Mtabolite | BBB permeability | P-gp substrate |
| --- | --- | --- |
| N1 | Yes | Yes |
| N4 | Yes | Yes |
| N6 | Yes | Yes |
| N8 | Yes | Yes |
